# Clinical validation of a vial wall-based dielectric barrier discharge plasma activation device for plasma-activated platelet-rich plasma preparation in osteoarthritis pain management

**DOI:** 10.1016/j.mtbio.2026.103766

**Published:** 2026-10-20

**Authors:** Shengfang Wang, Zhipeng Li, Zunlei Gong, Zengfa Deng, Zhihao Jin, Bo Pang, Jiawei Yao, Shuqin Zhang, Wenting Zhu, Jien Han, Yang Dai, Lianghao Wan, Jinglin Wan, Meijing Liu, Suan Tang, Li Jiang, Xiaogang Wang

**Affiliations:** a Guangdong Provincial Key Laboratory of Bone and Joint Degenerative Diseases, The Third Affiliated Hospital of Southern Medical University, Guangzhou, Guangdong, China; b State Key Laboratory of Multi-organ Injury Prevention and Treatment, Southern Medical University, Guangzhou, China; c Department of Rehabilitation Medicine, The Third Affiliated Hospital of Southern Medical University, Guangzhou, China; d Department of Sports Medicine, Center for Orthopaedic Surgery, The Third Affiliated Hospital of Southern Medical University, Guangzhou, Guangdong, China; e Clinical Research Centre, Zhujiang Hospital of Southern Medical University, Guangzhou, Guangdong, China; f School of Materials Science and Engineering, South China University of Technology, Guangzhou, Guangdong, China; g National Engineering Research Center for Tissue Restoration and Reconstruction, South China University of Technology, Guangzhou, Guangdong, China; h Corona Lab, Nanjing Suman Plasma Technology Co., Ltd, Nanjing, Jiangsu, China; i Department of Spinal Surgery, Orthopedic Medical Center, Zhujiang Hospital of Southern Medical University, Guangzhou, China; j Institute of Exercise and Rehabilitation Science, Zhujiang Hospital of Southern Medical University, Guangzhou, China

## Abstract

Cold atmospheric plasma (CAP) has significant potential in biomedical applications. However, conventional plasma activation requires electrodes to contact a liquid directly, exposing it to the external environment. This conflicts with sterile, closed preparations required clinically. A vial wall-based dielectric barrier discharge (DBD) plasma activation device addresses this need by using standard clinical blood collection tube walls as a dielectric barrier. Electrodes contact only the tube exterior, never the liquid inside, and activation requires simply inserting the tube into the reactor chamber. Applied to platelet-rich plasma (PRP) therapy for osteoarthritis (OA), the device produced plasma-activated PRP (PA-PRP) with enhanced platelet activation, increased levels of transforming growth factor-β (TGF-β; 24%), platelet-derived growth factor (PDGF; 37%), and insulin-like growth factor-1 (IGF-1; 32%), and broadly upregulated anti-inflammatory cytokines. In the destabilization of the medial meniscus (DMM) mouse OA model, PA-PRP treatment was associated with improved gait parameters and lower cartilage degeneration scores relative to PRP treatment. Human OA cartilage explants showed greater matrix staining after PA-PRP treatment. In an exploratory, randomized, single-blind split-body study involving three patients, PA-PRP-treated knees, compared with contralateral PRP-treated knees, showed numerically larger improvements in visual analog scale (VAS) pain score, Western Ontario and McMaster Universities Osteoarthritis Index (WOMAC) score, and effusion volume at week 15, with no adverse events during observation. These clinical observations are descriptive and hypothesis-generating, as the sample size precludes reliable efficacy or comparative safety inferences. Together, findings support device feasibility and PA-PRP use in OA and provide a rationale for adequately powered studies.

## Highlights

• A vial wall-based dielectric barrier discharge device activates platelet-rich plasma in sealed tubes.
• The tube-sealed workflow showed lower contamination risk than open plasma activation.
• Plasma activation increased platelet activation markers and growth-factor concentrations.
• Plasma-activated platelet-rich plasma was associated with improved outcomes in exploratory preclinical models.
• Three-patient clinical observations were descriptive and support future adequately powered trials.

## Graphical abstract

**Figure:**
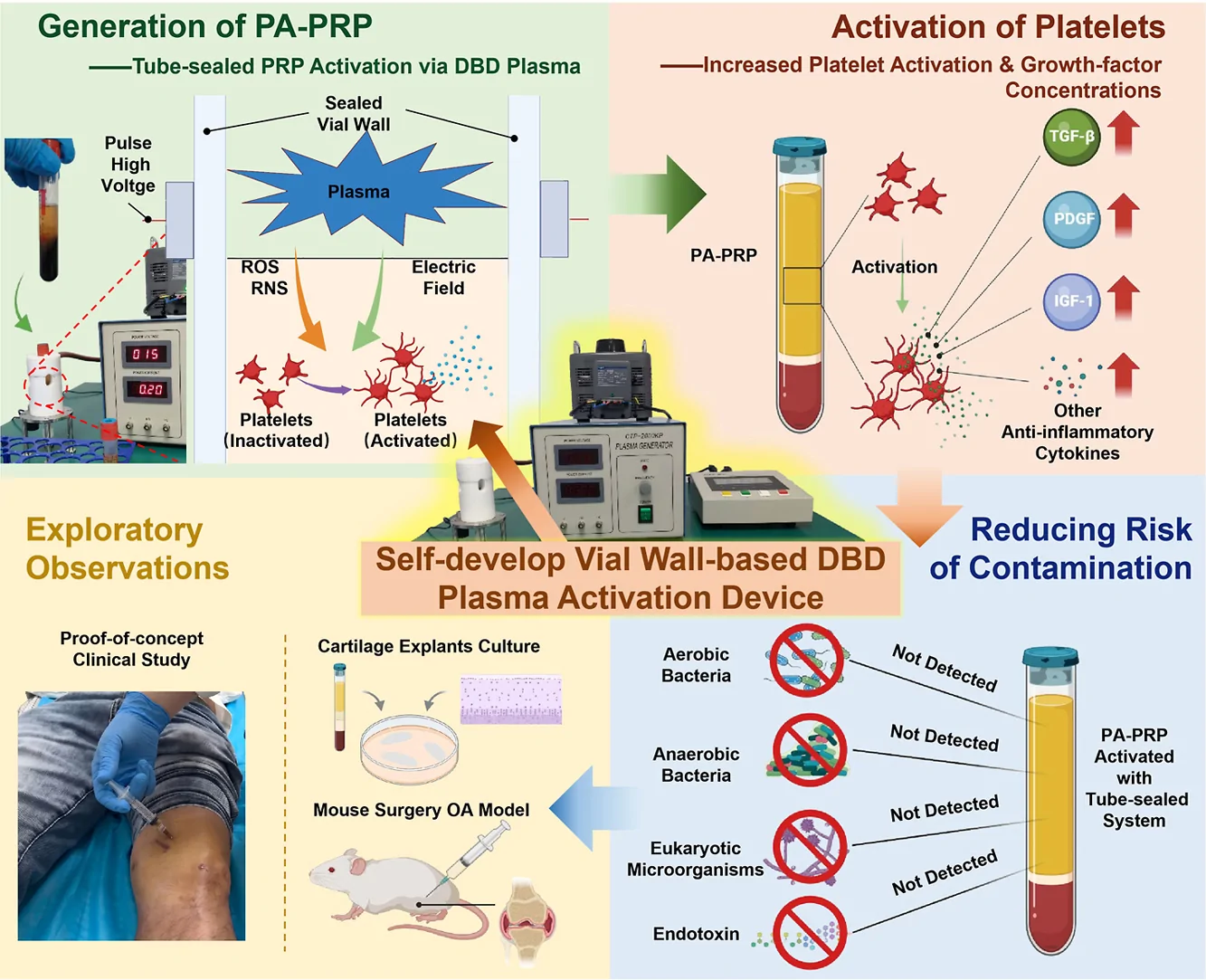

## 1 Introduction

Cold atmospheric plasma (CAP) has attracted substantial interest as a biophysical intervention for diseases such as osteoarthritis (OA) and periodontitis due to its ability to generate a localized electric field and a physicochemical environment rich in reactive oxygen species (ROS), reactive nitrogen species (RNS), electrons, and ions under ambient conditions [1], [2]. Studies on the biological effects of CAP-derived active components have progressively clarified that plasma-generated ROS and electric fields function as signaling factor that modulate tissue cell phenotype and function [2], [3], [4] and drive macrophage polarization toward the anti-inflammatory, pro-repair M2 subtype through defined signaling pathways [5]. Despite the mechanistic promise of CAP, it is largely confined to preclinical laboratory research, and its translation into routine clinical use is not yet realized. A principal barrier to its more widespread use is the limited penetration depth of plasma in biological tissue [6], [7]. This has motivated the development of injectable plasma-activated liquid products to broaden the therapeutic scope of CAP to deep anatomical sites, such as the articular cavity [2]. Most laboratory studies on plasma activation devices have used open-operation designs, such as plasma jets or discharge chambers [8], [9], [10], in which the target liquid sample is activated through direct contact with the plasma source. While these methods are straightforward to implement in exploratory research, the open design of the methods are inherently incompatible with the sterility, standardization, and quality control requirements of clinical preparation.

Platelet-rich plasma (PRP) therapy provides a clinical context in which CAP-based activation may address a recognized unmet need. OA affects over 100 million people globally and imposes a substantial burden on public health systems [11], [12]. PRP has become a widely adopted treatment option for knee OA, with clinical evidence confirming that it provides meaningful pain relief [13], [14], [15]. Efforts to optimize PRP efficacy have included improving platelet recovery rates [16], [17], increasing the platelet concentration [18], [19], and introducing exogenous chemical activators such as thrombin or calcium chloride [20], [21]. Nevertheless, clinical outcomes remain variable, with some patients experiencing suboptimal benefit [22], [23]. Insufficient platelet activation with incomplete growth factor release is considered a potential contributor to this inconsistency [24], [25]. A standardized, contamination-free activation method that enhances platelet degranulation and growth factor release while meeting the sterility standards required of an injectable therapeutic would therefore address a clear clinical need. CAP, with its ability to induce endogenous platelet signaling via ROS-mediated pathways [26], [27], is well-positioned to fill this need.

The device design reported here is based on a deliberate engineering decision to repurpose existing clinical equipment as functional device components. Standard clinical blood collection tubes, used universally in PRP preparation, have high dielectric strength, cylindrical symmetry, hermetic sealing, and pre-sterilization properties that map directly to the dielectric barrier requirements of DBD plasma generation. We therefore designed and constructed a fully enclosed, non-contact prototype for plasma activation in which the blood collection tube wall serves as an integral dielectric barrier. We refer to this as a vial wall-based DBD plasma activation system. In this configuration, high voltage and ground electrodes are positioned externally and adapted to the tube wall, and plasma is generated within the headspace of the liquid within the sealed tube without physically breaching the container. The liquid in the sealed tube is activated through the tube wall by the plasma field, thus preserving the original sterility of the sample throughout the process. This design eliminates the need for open sample handling inherent to conventional plasma activation workflows, simplifies the preparation procedure, and provides activation based on standardized parameters that is independent of operator techniques. These features collectively resolve many translation barriers limiting the clinical adoption of plasma medicine.

We evaluated the device by performing technical characterizations; paired PRP assays; cell-based, mouse OA model, and human OA cartilage explant experiments; and an exploratory split-body clinical study. The plasma-activated PRP (PA-PRP) produced using this device was associated with platelet activation, high growth factor concentrations, and macrophage marker changes. Pharmacological ROS scavenging attenuated the PA-PRP–associated changes in the macrophage markers, which supported ROS involvement but did not identify the responsible reactive species or downstream pathways. We therefore examined a proposed combination of growth factor release and local immunomodulation as a mechanistic hypothesis. The clinical component, in which three patients were evaluated, was designed to assess procedural feasibility and generate hypotheses for a future adequately powered randomized trial.

## 2 Results

### 2.1 Sealed-tube plasma activation via DBD using medical blood collection tubes as a dielectric barrier

We designed and constructed a vial wall-basedDBD plasma activation system using the wall of standard clinical blood collection tubes as a natural dielectric barrier to achieve non-contact, sterile activation of liquid samples within sealed commercial blood collection tubes. The system comprises a pulsed power supply, a direct current-to-alternating current (DC-AC) high-voltage converter, a pulse controller, and a vial wall-based DBD reactor (Fig. 1A). The entire circuit is connected to a standard ground terminal. The assembled prototype device was integrated according to the design schematic shown in Fig. 1B, with the vial wall-based DBD reactor suitable for holding sealed blood collection tubes. As illustrated in the reactor schematic (Fig. 1C), high-voltage and grounded electrodes are arranged on the outer wall of the sealed blood collection tube, with the high-impedance vial wall of the collection tube directly functioning as the dielectric barrier for DBD discharge. Plasma is generated in the sealed tube in the headspace above the liquid after the high-voltage pulsed power supply is switched on and a stable operating voltage is reached (Fig. 1C). Under an input power of 3 W, plasma corona discharge was observed as a blue filamentary discharge in the headspace above the PRP liquid within the sealed blood collection tube (Fig. 1C). This input was found to be the minimum sustaining condition of the system. An oscilloscope showed that the peak output voltage and current at the minimum sustaining power were approximately 6.08 kV and 7.45 mA, respectively, with a frequency of 10 kHz (Fig. 1D and E). With a 0.47-μF sampling capacitor at the interface to measure the current, a Lissajous figure was drawn by integrating the output charge per cycle and output voltage, with the output power calculated as 1.58 ± 0.18 W via the formula $P=\frac{1}{T}\int_{0}^{T}U\left(t\right)I\left(t\right)dt$. This corresponded to a discharge efficiency of 52.57% ± 6.05% with an input power of 3 W (Fig. 1F and G; n = 3 independent experiments).

**Fig. 1:**
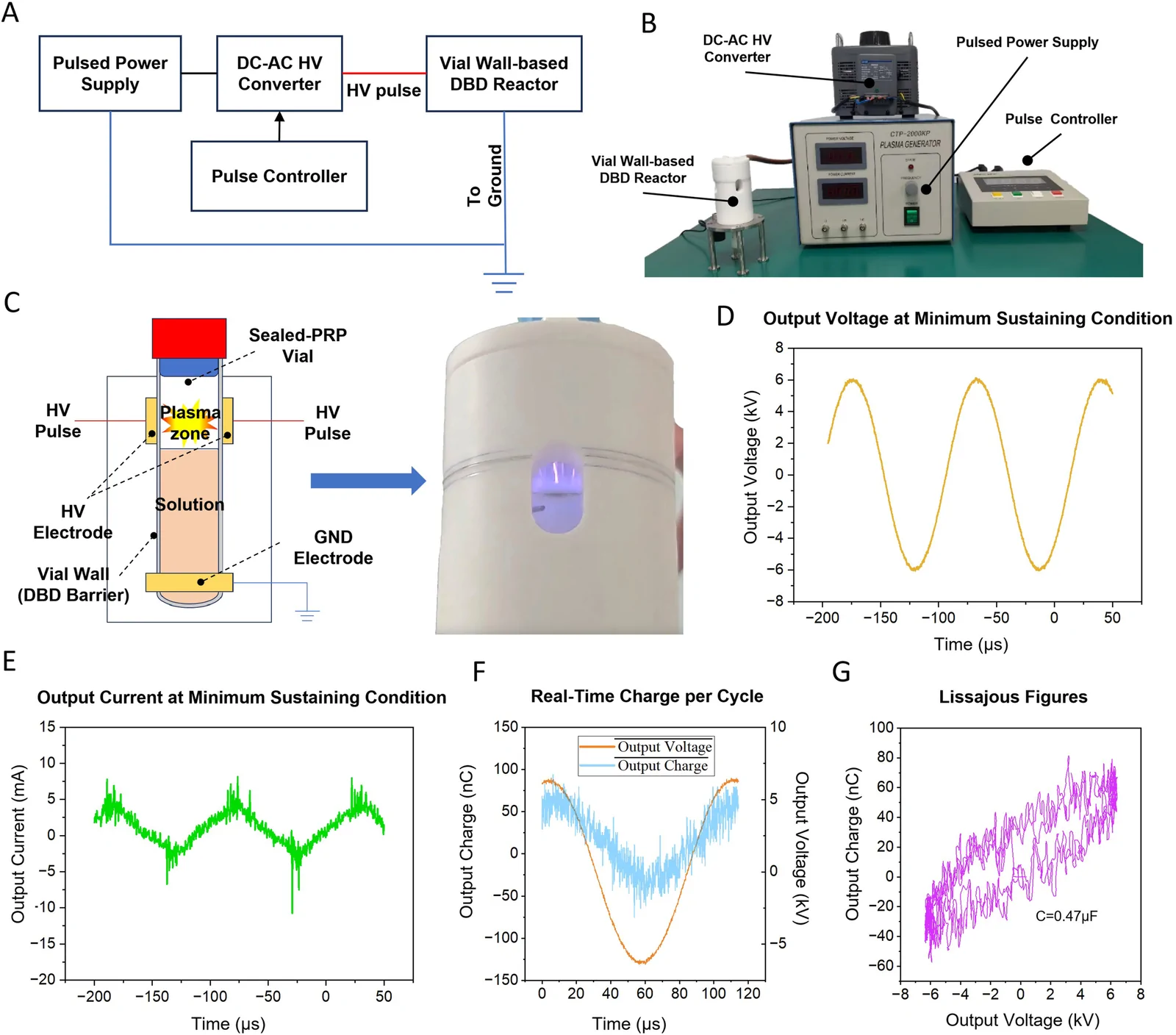
**Discharge characteristics of the self-developed vial wall-based DBD plasma activation device.** A. Schematic circuit diagram of the self-developed vial-wall based DBD plasma activation device, illustrating its key electronic components. B. Photograph of the prototype for the self-developed plasma activation device, which operates on the DBD principle using the vial wall of a blood collection tube as the dielectric barrier. C. Schematic diagrams of the vial wall-based DBD reactor illustrating its structure and the discharge process. D. Representative data showing the waveforms of the pulsed output voltage at minimum sustaining condition of this device. E. Representative data showing the waveforms of the pulsed output current at minimum sustaining condition of this device. F. Representative data showing the waveforms of the real-time charge each cycle at minimum sustaining condition of this device (C = 0.47 μF) G. Representative data showing the Lissajous figures during minimum sustaining condition of this device.

Commercially available blood collection tubes from different brands vary in their material, wall thickness, intratubal negative pressure, relative permittivity, and other properties that may affect the discharge. We tested five common brands and verified that all the blood collection tubes achieved stable corona discharge with this device. By using the increase in the ROS concentration as the indicator for successful PA-PRP preparation, all tested brands produced qualified PA-PRP under identical activation parameters (Supplementary Table 1).

### 2.2 Conditions for controlled and reproducible activation of PRP with reduced contamination risks

The input parameters of the proposed device can be precisely regulated to control the PRP activation conditions. Under identical input parameters, the temperature of the activated product increased as the activation duration increased. Increasing the input power or duty cycle also led to a rise in the product temperature. When PRP activation was performed at an input power of 6 W and a 1% duty cycle for 15 s, the final temperature of the product reached 36.7 ± 1.5°C (Fig. 2A; n = 3‌ independent repeats). Increasing either the duty cycle or activation duration both gradually decreased the product pH. Inactivated PRP has a pH consistent with that of whole blood, and its pH ranges from 7.2 to 7.4. After activation using these settings (6 W, 1% duty cycle, 15 s), the pH decreased slightly to 7.307 ± 0.031 (Fig. 2B; n = 3 independent repeats), which remained within the physiological range. However, when the activation duration exceeded 30 s at this duty cycle, the pH dropped sharply beyond the buffering capacity of the PRP. Increasing the duty cycle or extending the activation duration also significantly increased the ROS concentration in the activated product (Fig. 2C; n = 3 independent repeats). To preserve the bioactivity of the PRP components and maintain activation within physiological ranges, we defined the optimal parameter window to achieve a final temperature no higher than 37°C, with pH maintained between 7.2 and 7.4. Therefore, the appropriate activation parameter setting were determined to be as follows: input power 6 W, duty cycle 1%, and activation duration 15 s. Using these settings, the output power calculated from the Lissajous figure obtained via oscilloscope measurements was approximately 3.12 ± 0.19 W (Supplementary Fig. 1C), corresponding to about 52.06% ± 3.20% of the input power. This ratio was consistent with the output power ratio observed under the minimum sustaining power (Fig. 1G). Using the self-developed vial wall-based DBD plasma activation device with optimized parameters (input power of 6 W, duty cycle 1%, and activation duration 15 s), 50 consecutive saline samples were activated using a single brand of blood collection tubes. ROS concentrations measured using a commercial kit showed that the differences across the 50 samples were minimal, with a coefficient of variation (CV) of only 4.53% (Fig. 2D), which confirmed the reliable working stability of the device for continuous operation.

**Fig. 2:**
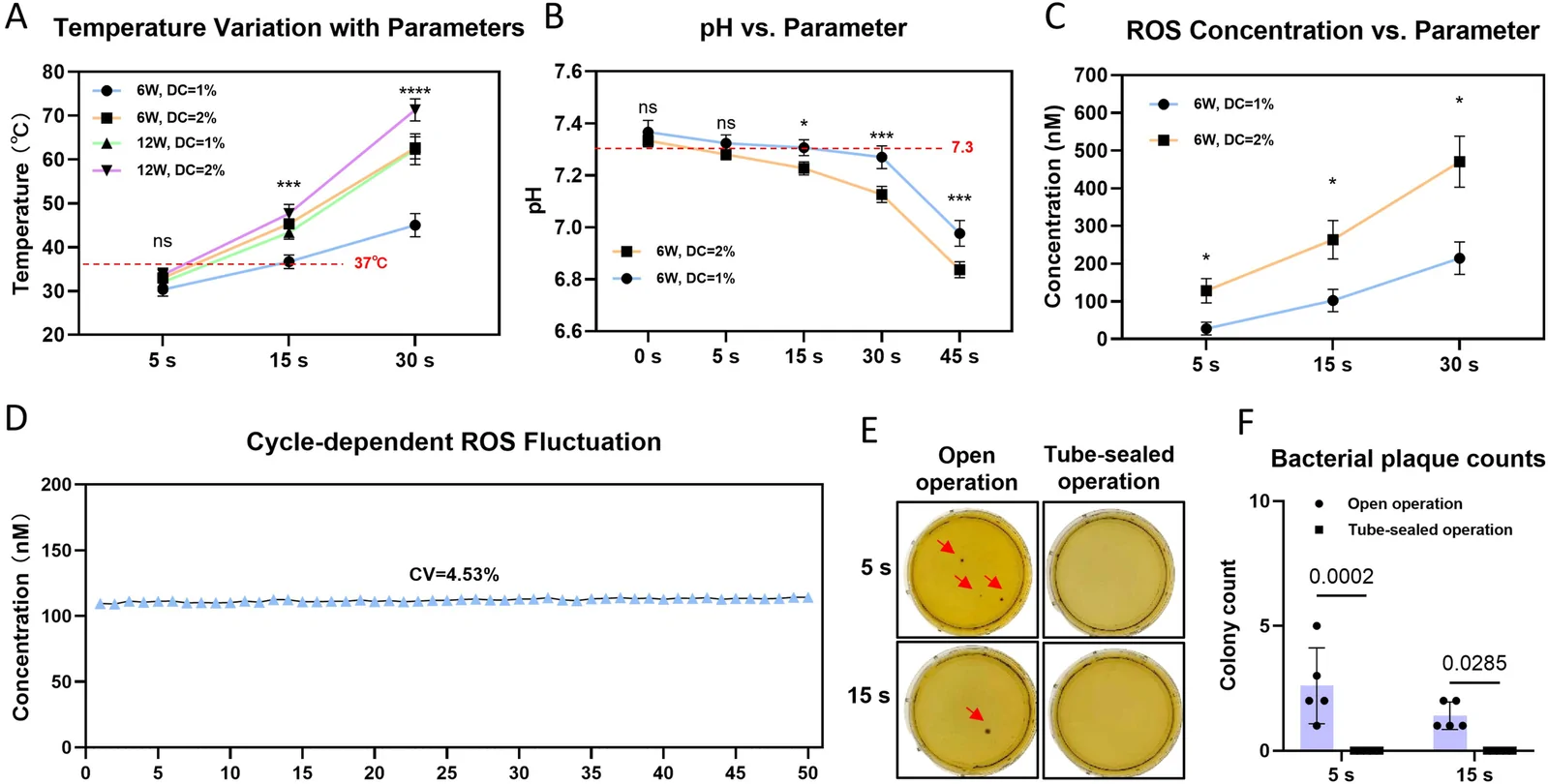
**Tunability, stability, and sterility of the plasma activation process.** A. Line graph showing the temperature changes of plasma-activated liquids generated under different device parameters (n = 3 independent experiments; data are presented as mean ± SD, analyzed by two-way ANOVA; *F* (3, 24) = 65.29; **** represent p < 0.0001; *** represent p < 0.001; ns represent no significant difference). B. Line graph showing the pH changes of plasma-activated liquids generated under different device parameters (n = 3 independent experiments; data are presented as mean ± SD, analyzed by two-way ANOVA; *F* (1, 4) = 45.31; ** represent p < 0.01; *** represent p < 0.001; ns represent no significant difference). C. Line graph showing the concentration changes of reactive oxygen species (ROS) in plasma-activated liquids generated under different device parameters (n = 3 independent experiments; data are presented as mean ± SD, analyzed by two-way ANOVA; *F* (1, 4) = 44.46; * represent p < 0.05). D. Line graph showing the trend of ROS concentration in 50 sequentially prepared plasma-activated liquid samples, demonstrating the stability over 50 activation cycles. E. Representative image comparing the contamination status after the "tube-sealed activation" using this device versus the conventional open-air activation process (red arrows indicate bacterial colonies). F. Bar graph comparing the number of bacterial colonies resulting from the "tube-sealed activation" versus the open-air activation (n = 5 independent experiments; data are presented as mean ± SD; analyzed by two-way ANOVA, followed by paired Student's *t*-tests for within-group comparisons where applicable; F (1, 8) = 40.00; *p* values are shown). (For interpretation of the references to color in this figure legend, the reader is referred to the Web version of this article.)

A contamination risk assessment was conducted to confirm the intended safety functions of the tube-sealed activation. PA-PRP samples activated by the device were extracted from the sealed blood collection tubes, and contamination was evaluated by multiple classes of microorganisms and endotoxin. PA-PRP samples were inoculated onto Luria-Bertani (LB) agar (aerobic bacteria), Sabouraud dextrose agar (eukaryotic microorganisms, including fungi and yeasts), and Columbia blood agar (anaerobic bacteria) for standard culturing, and the endotoxin concentration was quantified via a chromogenic limulus amebocyte lysate (LAL) assay. PA-PRP samples activated by the device for 5 s or for 15 s were extracted from the sealed blood collection tubes and spread onto LB agar plates for a 24-h incubation at 37°C. No bacterial colonies were observed on any of the plates (Fig. 2E and F). Consistent with the aerobic bacteria testing results, no colonies of eukaryotic microorganisms or anaerobic bacteria were detected in the PA-PRP samples activated by the device after an appropriate incubation period (Supplementary Fig. 2A). In contrast, under identical parameters, obvious eukaryotic microbial colonies were present on Sabouraud dextrose agar plates spread with PA-PRP activated by a conventional open plasma jet device, which required the tube seal to be broken for direct contact between the plasma jet and the liquid surface (Fig. 2E and F and Supplementary Fig. 2A). No anaerobic colonies were observed in either group, consistent with the lower abundance of anaerobes in ambient air (Supplementary Fig. 2A). Furthermore, endotoxin contamination testing showed that all tube-sealed PA-PRP samples activated by the device tested below the limit of detection, while PA-PRP prepared by the conventional open operation method contained detectable trace endotoxin ranging from 200 pg/mL to 1500 pg/mL across replicates (Supplementary Fig. 2B). This result confirmed that our proposed tube-sealed activation device significantly reduced the risk of microbial and endotoxin contamination compared with a conventional open plasma activation system.

### 2.3 Successful preparation of plasma-activated PRP using the device

Human PRP was isolated from peripheral venous blood following a standard clinical protocol, with the entire process completed within the sealed commercial blood collection tube. After preparing the PRP, the entire sealed blood collection tube was directly inserted into the vial wall-based DBD reactor of our device. By operating the device using the optimized parameters (6 W input power, duty cycle 1%, and activation duration 15 s), PA-PRP was obtained (Fig. 3A). Immunofluorescence staining showed that after plasma activation, 43.04% ± 9.67% of cluster of differentiation 41 (CD41)-positive platelets in PA-PRP expressed the activation marker cluster of differentiation 63 (CD63), which was a significantly higher percentage than in PRP (Fig. 3B and C; n = 5 independent samples; *p* = 0.008, *t* = 5.265, df = 8), indicating that plasma activation successfully induced platelet activation. An enzyme-linked immunosorbent assay (ELISA) quantification of paired PRP and PA-PRP aliquots showed that the concentrations of TGF-β, total PDGF, and IGF-1 were higher after plasma activation (Fig. 3D–F; n = 5 pairs; two-tailed paired *t* tests, *p* = 0.0256, 0.0022, and 0.0096, respectively). A cytokine microarray analysis found that the concentrations of multiple cytokines and growth factors, including platelet-derived growth factor-BB (PDGF-BB), vitamin D-binding protein (DBP), vascular endothelial growth factor (VEGF), interleukin-10 (IL-10), insulin-like growth factor binding protein-2 (IGFBP-2), insulin-like growth factor binding protein-3 (IGFBP-3), interleukin 1 receptor antagonist (IL-1Ra), platelet-derived growth factor-AA (PDGF-AA), and interleukin-4 (IL-4), were all higher to varying degrees in the PA-PRP than in the PRP (Fig. 3G), further confirming that the plasma treatment stimulated the platelets and other cellular components in PRP to secrete bioactive factors. The cytological biological effects of the PA-PRP were further confirmed by culturing macrophages and chondrocytes in cell culture medium supplemented with 10% (v/v) PA-PRP. Compared with the culture containing PRP, the culture containing PA-PRP had lower expression of the M1 marker inducible nitric oxide synthase (*iNOS*) and higher expression of the M2 markers cluster of differentiation 206 (*CD206*) and arginase 1 (*ARG1*). Adding 10 mM N-acetylcysteine (NAC) to the PA-PRP–supplemented culture attenuated the changes in *iNOS* and *ARG1* expression (Supplementary Fig. 3). The phenotype and function of chondrocytes were positively maintained by the PA-PRP–supplemented culture (Supplementary Fig. 4).

**Fig. 3:**
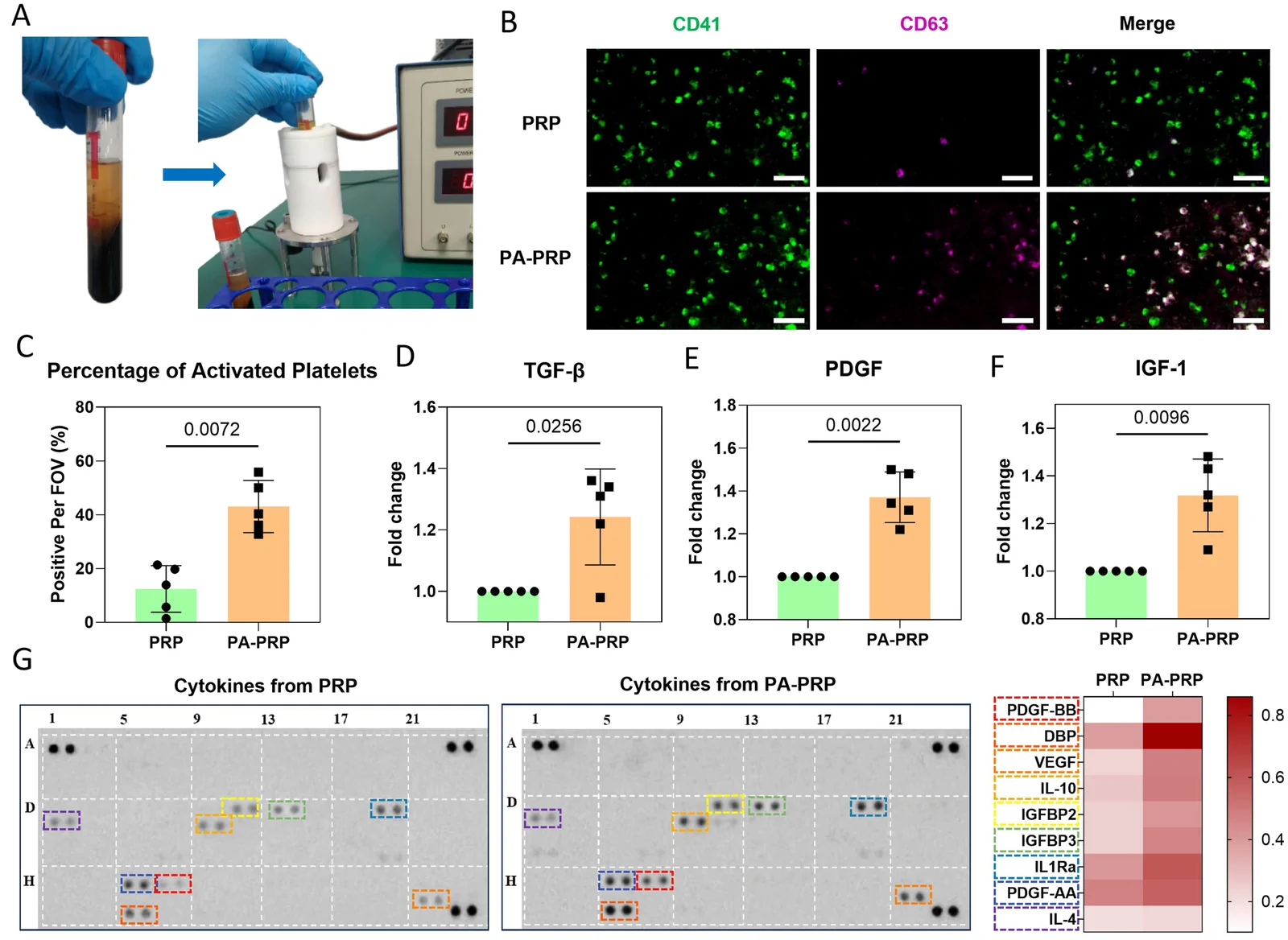
**Plasma activation enhances platelet activation and growth factors released from PRP.** A. Schematic diagram illustrating the procedure for plasma activation of PRP. B. Immunofluorescence images showing the activation status of platelets in PRP before and after plasma activation. Platelets are labeled with CD41 (pan marker, green) and CD63 (the activation marker, purple); scale bar = 5 μm C. Bar graph quantifying the percentage of activated platelets per field of view before and after plasma activation (quantified using ImageJ software; n = 5 independent samples each group; data are presented as mean% ± SD%; statistical analyzed by Student's *t*-test; *p* value is shown; *t* = 5.265; df = 8; 95% CI 17.20 to 44.01). D. Bar graph showing the relative concentration of TGF-β in paired PRP and PA-PRP aliquots, measured by ELISA (n = 5 pairs; mean ± SD; two-tailed paired *t*-test, *t* (4) = 3.470, unadjusted *p* = 0.0256; mean paired difference, 0.2420; 95% CI 0.0484 to 0.4356). E. Bar graph showing the relative concentration of total PDGF in paired PRP and PA-PRP aliquots, measured by ELISA (n = 5 pairs; mean ± SD; two-tailed paired *t*-test, *t* (4) = 7.015, unadjusted *p* = 0.0022; mean paired difference, 0.3706; 95% CI 0.2239 to 0.5173). F. Bar graph showing the relative concentration of IGF-1 in paired PRP and PA-PRP aliquots, measured by ELISA (n = 5 pairs; mean ± SD; two-tailed paired *t*-test, *t* (4) = 4.656, unadjusted *p* = 0.0096; mean paired difference, 0.3182; 95% CI 0.1285 to 0.5079). G. Representative image from a cytokine microarray, displaying the global changes in cytokine profiles after plasma activation of PRP. Cytokines with increased concentrations are highlighted within the dashed boxes and displayed with heat map. (For interpretation of the references to color in this figure legend, the reader is referred to the Web version of this article.)

### 2.4 PA-PRP effectively improves gait and alleviates articular cartilage damage in OA mice

A mouse OA model was established by destabilization of the medial meniscus (DMM) surgery on one hind limb, with sham surgery performed as the control. OA mice received intra-articular injections of PA-PRP or PRP once weekly starting the fifth week after DMM surgery for a total of four injections. Two weeks after the fourth injection (10th week post-surgery), a gait assessment was performed and joint tissue was harvested for histological staining (Fig. 4A). The body weight and survival of the mice in each group were monitored before each injection starting with the first administration of PA-PRP or PRP.

**Fig. 4:**
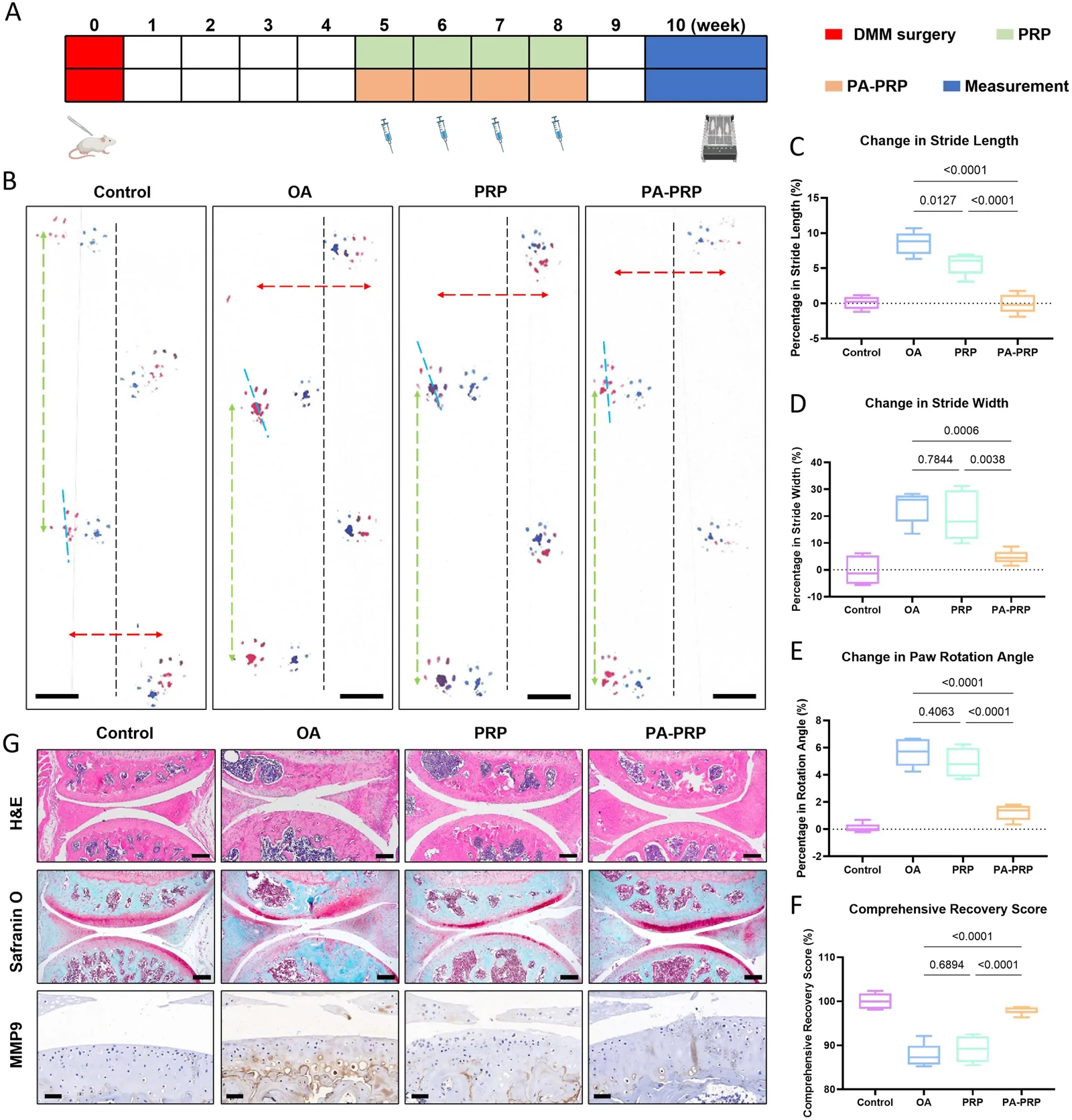
**PA-PRP treatment improves gait and alleviates cartilage damage in a mouse OA model.** A. Schematic diagram illustrating the experimental timeline and procedure for establishing the surgical osteoarthritis (OA) mouse model and subsequent PA-PRP treatment to evaluate therapeutic efficacy. B. Representative image of footprints from OA mice at the 10th week (the affected left hind paw is shown; hind paw prints are shown in red; fore paw prints are shown in blue; black dashed line indicates animal midline; green arrow indicates stride length; red arrow indicates stride width; blue dashed line indicates the direction of hind paw rotation; scale bar = 1 cm) C. Box plots showing percentage changes in stride length after treatment (n = 5/group; mean ± SD). Ordinary one-way ANOVA, *F* (3,16) = 46.90, *p* < 0.0001, followed by Šidák-adjusted multiple comparisons: OA versus PA-PRP, *p*_*adj*_ < 0.0001; OA versus PRP, *p*_*adj*_ = 0.0127; PRP versus PA-PRP, *p*_*adj*_ < 0.0001. D. Box plots showing percentage changes in hind-limb stride width after treatment (n = 5/group; mean ± SD). Ordinary one-way ANOVA, *F* (3,16) = 17.08, *p* < 0.0001, followed by Šidák-adjusted multiple comparisons: OA versus PA-PRP, *p*_*adj*_ = 0.0006; OA versus PRP, *p*_*adj*_ = 0.7844; PRP versus PA-PRP, *p*_*adj*_ = 0.0038. E. Box plots showing percentage changes in paw rotation angle after treatment (n = 5/group; mean ± SD). Ordinary one-way ANOVA, *F* (3,16) = 55.64, *p* < 0.0001, followed by Šidák-adjusted multiple comparisons: OA versus PA-PRP, *p*_*adj*_ < 0.0001; OA versus PRP, *p*_*adj*_ = 0.4063; PRP versus PA-PRP, *p*_*adj*_ < 0.0001. F. Box plots showing the composite gait-recovery index after treatment (n = 5/group; mean ± SD). The reported comparison between PRP and PA-PRP was originally generated as a nominal comparison (*t* = 6.266, *df* = 16, *p* < 0.0001). G. Histological staining results of knee joints from OA mice treated with PRP or PA-PRP; H&E and Safranin O-Fast Green staining images are shown at 20× magnification (scale bar = 100 μm). Immunohistochemical staining for MMP9 is shown at 40× magnification (scale bar = 50 μm). (For interpretation of the references to color in this figure legend, the reader is referred to the Web version of this article.)

The gait analysis of the DMM-induced OA mice revealed that, compared with the healthy hind limb, the contralateral destabilized limb exhibited a shortened stride length, increased hind limb stride width, and enlarged paw rotation angle (Fig. 4B–E), confirming that OA-related pain caused significant gait abnormalities. Stride length, stride width, and paw rotation angle differed among the groups (control, untreated OA, PRP, PA-PRP) by one-way ANOVA (all *p* < 0.0001; n = 5 per group). Šidák-adjusted comparisons found differences between the PA-PRP group and both the OA and PRP groups across all three gait parameters (Fig. 4B–E). The PRP group differed from the OA group in stride length (*p*_*adj*_ = 0.0127) but not in stride width or paw rotation angle (*p*_*adj*_ = 0.7844 and 0.4063, respectively). The composite gait recovery index was higher in the PA-PRP group than in the PRP group (97.69% ± 1.36% versus 86.35% ± 4.35%; Fig. 4F; nominal *p* < 0.0001). Note that these preclinical gait differences do not establish analgesic efficacy or a mechanism underlying the observed change.

Histological staining of the mouse knee joints confirmed severe articular cartilage damage, and Osteoarthritis Research Society International (OARSI) pathological scoring confirmed that PA-PRP treatment was associated with significantly lower cartilage degeneration scores than both untreated OA and PRP treatment (Fig. 4G and Supplementary Fig. 5B). Consistently, the levels of matrix-degrading enzymes were lower in the PA-PRP group, including matrix metalloproteinase 9 (MMP9), which was significantly lower (Fig. 4G), and matrix metalloproteinase 3 (MMP3) and 13 (MMP13) (Supplementary Fig. 5A). These results collectively suggest that PA-PRP has the potential to delay articular cartilage degeneration in OA.

### 2.5 PA-PRP is associated with higher matrix-staining signals in human osteoarthritic cartilage explants *ex vivo*

Human osteoarthritic cartilage explants were harvested from discarded OA knee tissue and cultured in basal medium supplemented with PRP or PA-PRP for 14 days to further examine the direct chondroprotective effect of PA-PRP and confirm the findings from our mouse OA model. Representative histological images showed that the control cartilage explants exhibited obvious loss of the extracellular matrix, while PA-PRP treatment restored more of the cartilage matrix than PRP treatment (Fig. 5A). A quantitative analysis of the normalized intensity of Safranin O staining, a marker of cartilage proteoglycan content, confirmed that PA-PRP significantly increased the matrix staining intensity relative to the PRP and control groups (Fig. 5B). Consistent with this result, the normalized intensity of Alcian blue staining, another specific stain for acidic glycosaminoglycans in the cartilage matrix, was also significantly higher in the PA-PRP group than in the PRP and control groups (Fig. 5C). The *ex vivo* testing showed higher matrix-staining signals after PA-PRP treatment under the tested culture conditions and supported further investigation of possible chondroprotective activity.

**Fig. 5:**
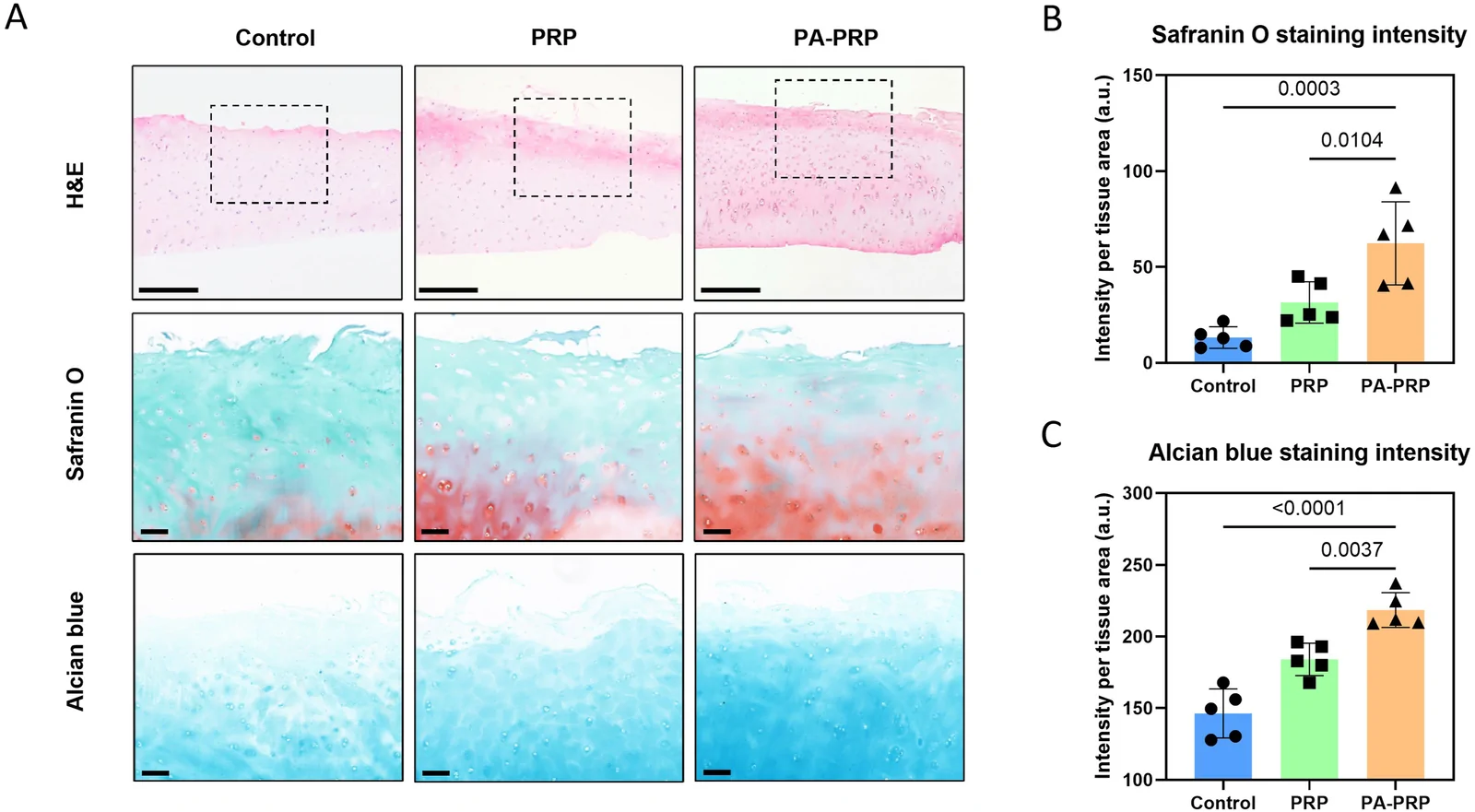
**PA-PRP treatment inhibits extracellular matrix degradation of ex *vivo* human osteoarthritic cartilage explants.** A. Representative histological staining images of cartilage explants treated with PRP or PA-PRP; H&E staining are shown at 4× magnification (scale bar = 500 μm); Safranin O-Fast Green staining and Alcian blue staining are shown at 20× magnification (scale bar = 100 μm) B. Bar graph displayed the quantitative analysis of normalized Safranin O staining intensity (n = 5/group independent samples; data are presented as mean% ± SD%; statistical analyzed by one-way ANOVA, followed by paired Student's *t*-tests for within-group comparisons where applicable; *p* values are shown; *F* (2, 12) = 15.05). C. Bar graph displayed the quantitative analysis of normalized Alcian blue staining intensity (n = 5/group independent samples; data are presented as mean% ± SD%; statistical analyzed by one-way ANOVA, followed by paired Student's *t*-tests for within-group comparisons where applicable; *p* values are shown; *F* (2, 12) = 34.57). (For interpretation of the references to color in this figure legend, the reader is referred to the Web version of this article.)

### 2.6 Exploratory clinical observations in patients with mild-to-moderate knee osteoarthritis

To further validate the early clinical efficacy of PA-PRP, we conducted a preliminary proof-of-concept clinical trial in three patients diagnosed with bilateral mild-to-moderate knee OA. Using a randomized, single-blind, split-body study design, each patient received intra-articular injections of PA-PRP in one randomly assigned knee and conventional PRP injections in the contralateral knee, with three injections administered once per week. A baseline assessment was performed before the first injection, which included ultrasound imaging, visual analog scale (VAS) pain scores, Western Ontario and McMaster Universities Osteoarthritis Index (WOMAC) scores, and knee flexion angle measurements. A follow-up VAS pain score assessment was performed 1 week after the first injection. All baseline assessments were repeated at the 15th week (Fig. 6A and B).

**Fig. 6:**
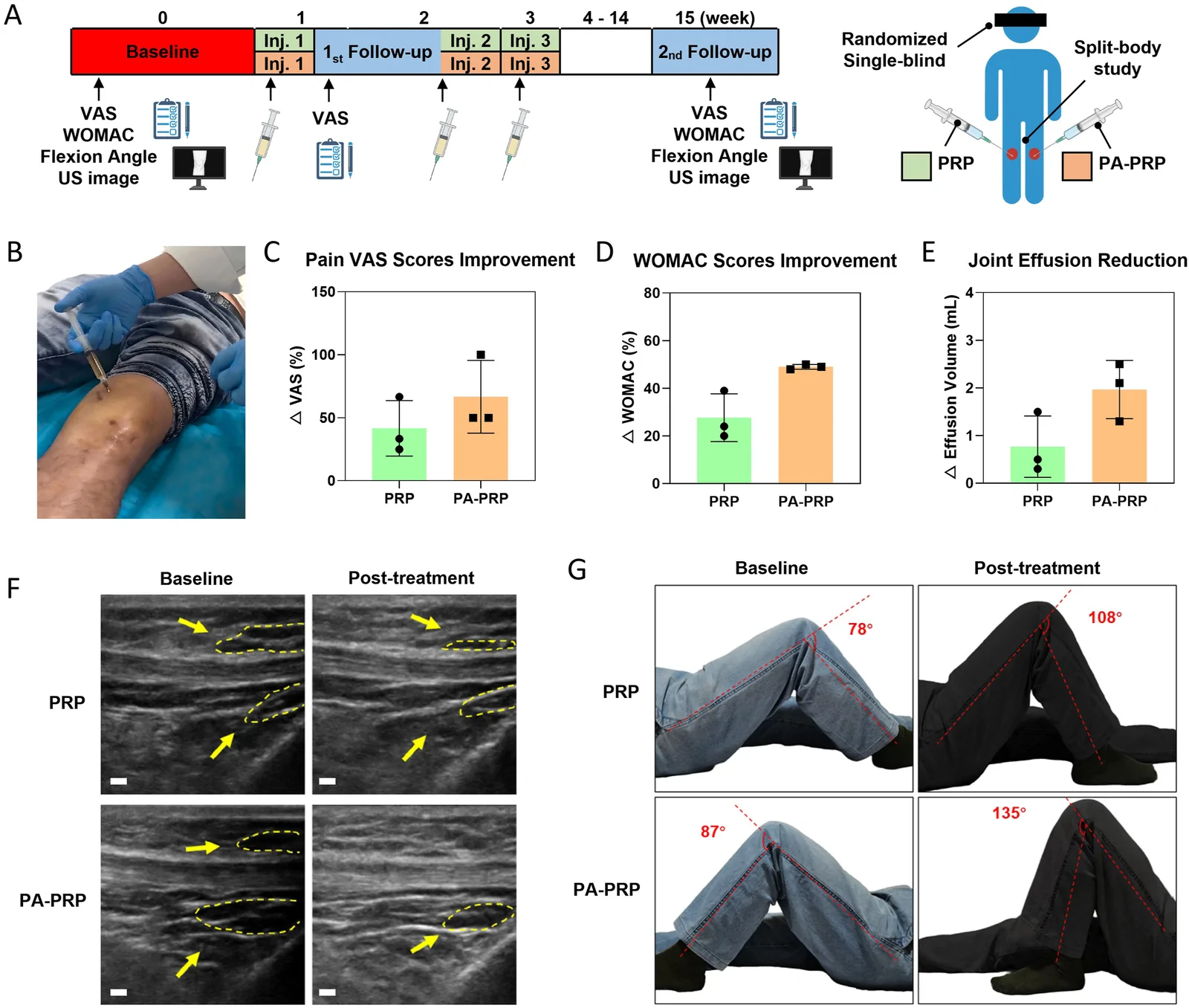
**Exploratory clinical observations of PA-PRP injection in patients with osteoarthritis.** A & B. Schematic of the exploratory clinical study protocol, showing the design and timeline of the exploratory preliminary proof-of-concept split-body, single-blind clinical study. C. Individual paired VAS observations and descriptive change from baseline to the week-15 follow-up in the three enrolled participants (No inferential statistical testing was performed). D. Improvement in the WOMAC score from baseline to last follow-up (No inferential statistical testing was performed). E. Individual paired observations of aspirated knee-effusion volume and descriptive mean ± SD change across the reported clinical time points in the three enrolled participants (No inferential statistical testing was performed). F. Representative B-mode ultrasound image of a patient's knee joint, with joint effusion highlighted within the yellow dashed area and indicated by a yellow arrow (scale bar = 1 mm) G. Representative images comparing the range of motion in knee flexion for a patient at baseline and at last follow-up. (For interpretation of the references to color in this figure legend, the reader is referred to the Web version of this article.)

At week 15, both treatments showed numerical improvements from baseline in the three enrolled participants. The PA-PRP–treated knees had larger descriptive mean changes in the VAS pain scores, total WOMAC scores, and aspirated effusion volumes than the contralateral PRP-treated knees. The VAS score reduction of the PA-PRP treatment compared with the PRP treatment was 72.20 ± 25.47% and 44.40 ± 19.23% (Fig. 6C), respectively; the WOMAC improvement was 49.00 ± 1.00% and 27.67 ± 10.02%, respectively (Fig. 6D; Table 2); and the effusion volume reduction was 1.97 ± 0.61 mL and 0.77 ± 0.64 mL, respectively (Fig. 6E). Paired ultrasound and flexion angle observations are presented in Fig. 6F and G and Table 3, and the early VAS observations are summarized in Table 1. No adverse events were recorded during the scheduled clinical follow-up visits (Table 4). The individual paired observations and descriptive summaries are presented without inferential hypothesis testing, confidence intervals, or statistical significance claims. The apparent early VAS pattern and between-knee numerical differences observed in these patients are feasibility observations that require assessment in an adequately powered randomized controlled trial with prespecified clinical endpoints.

**Table 1:** Comparison of VAS scores before and after treatment.

				PRP			PA-PRP		
	Sex	Age	K-L grade	Baseline	Week 2	Week 15	Baseline	Week 2	Week 15
Patient 1	M	65	3	6	4	4	6	4	3
Patient 2	M	33	2	4	3	3	4	2	2
Patient 3	F	45	2	3	2	1	3	2	0

**Table 2:** Changes in WOMAC scores from baseline to post-treatment.

	PRP		PA-PRP	
	Baseline	Week 15	Baseline	Week 15
Patient 1	27.00	16.67	27.00	13.54
Patient 2	25.00	18.75	25.00	13.00
Patient 3	6.25	5.00	6.25	3.13

**Table 3:** Changes in knee flexion angle degrees following treatment.

	PRP		PA-PRP	
	Baseline	Week 15	Baseline	Week 15
Patient 1	78°	108°	87°	135°
Patient 2	113°	136°	110°	145°
Patient 3	128°	136°	128°	149°

**Table 4:** No adverse events recorded.

	Treatment	Baseline	1st injection	Week 2	2nd injection	3rd injection	Week 15
Patient 1	PRP	None	None	None	None	None	None
	PA-PRP	None	None	None	None	None	None
Patient 2	PRP	None	None	None	None	None	None
	PA-PRP	None	None	None	None	None	None
Patient 3	PRP	None	None	None	None	None	None
	PA-PRP	None	None	None	None	None	None

### 2.7 Short-term biocompatibility and safety of PA-PRP

Short-term biocompatibility was assessed *in vitro* in chondrocytes and macrophages. Cell survival did not differ significantly among the control, PRP, and PA-PRP groups under the tested conditions (Fig. 7A and B). In the 10-week mouse study, all mice survived to the planned endpoint, the body weight gain did not differ detectably between the groups, and a histological examination of the heart, liver, spleen, lung, and kidney showed no overt pathological abnormalities under the tested conditions (Fig. 7C–E). These observations provide short-term *in vitro* and *in vivo* safety information.

**Fig. 7:**
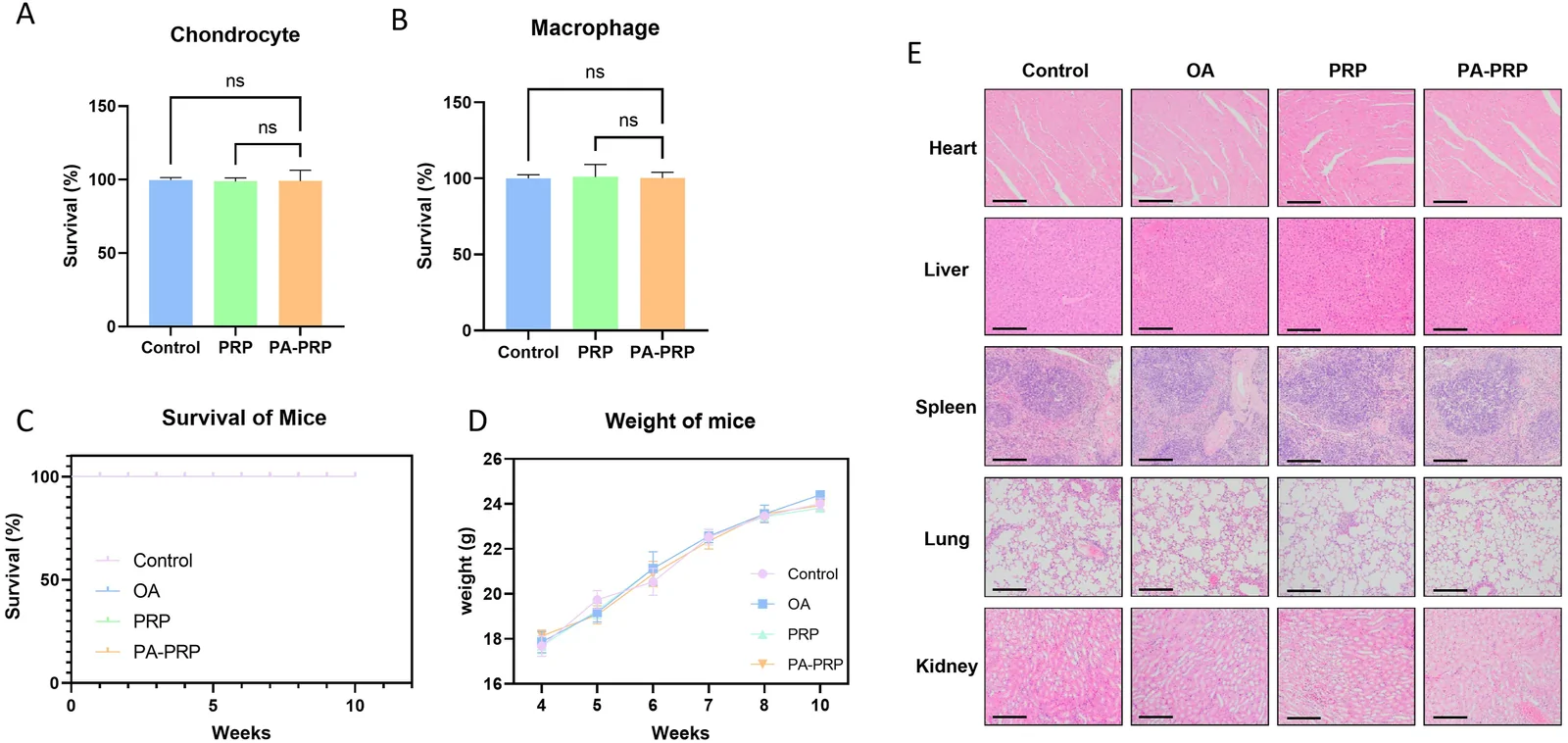
**PA-PRP demonstrates a safety profile both in *vitro* and in *vivo*.** A. Bar graph showing chondrocyte survival rate after culture with FBS, PRP, or PA-PRP (n = 3/group independent experiments; data are presented as mean ± SD; analyzed by one-way ANOVA followed by paired Student's *t*-tests for within-group comparisons where applicable; *F* (2, 6) = 1.000; ns = no significant difference). B. Bar graph showing macrophage survival rate after culture with control, PRP, or PA-PRP (n = 3/group independent experiments; data are presented as mean ± SD; analyzed by one-way ANOVA followed by paired Student's *t*-tests for within-group comparisons where applicable; *F* (2, 6) = 1.000; ns = no significant difference). C. Kaplan-Meier survival curves of mice in the control, OA, PRP, and PA-PRP groups over 10 weeks of observation (n = 5/group, data are presented as mean% ± SD%). D. Line graph showing changes in body weight of mice in the four study groups from week 4 to week 10 after modeling (n = 5/group, data are presented as mean ± SD). E. Representative H&E staining images of major organs shown at 40× magnification, which are heart, liver, spleen, lung and kidney, collected from mice in the four study groups at 10 weeks (scale bar = 200 μm).

In the three-patient split-body feasibility study, adverse events were recorded at each scheduled follow-up. No injection site reactions, infection, inflammatory reactions, or systemic adverse effects were reported in either treatment knee during the observation period (Table 4). This small, short-term observation supports the feasibility of a further safety evaluation, while long-term toxicology and repeated dose intra-articular safety require dedicated assessments in larger studies.

## 3 Discussion

In addition to meeting sterility requirements, a clinically viable plasma activation device must demonstrate electrical stability, batch-to-batch reproducibility, and robustness to variability in consumables. These characteristics must be established before biological or clinical validation can be meaningfully interpreted. The electrical characterization of the vial wall-based DBD device confirmed that standard clinical blood collection tubes function as effective dielectric barriers during plasma generation. The power transfer efficiency remained at approximately 52% under both the minimum sustaining power and optimized parameter conditions. This consistency indicated that the discharge behavior was governed primarily by the fixed geometry and material properties of the tube wall. In testing with 50 consecutive activations, the CV in the ROS concentration was 4.53%. The 50 experimental runs provided an initial estimate of the reproducibility of the system during continuous operation. The device also generated a consistent discharge when tested with different commercial blood collection tubes, despite differences in the tube wall material, thickness, and dielectric constant. Broader compatibility, lot-to-lot reproducibility, and manufacturing quality control will require dedicated validation before routine clinical deployment of the device.

The sterility advantage of the sealed-tube design provides a categorical improvement in safety. No bacterial colonies were detected after a 24-h incubation of the PA-PRP activated by the device, whereas colonies were consistently observed in PA-PRP activated by a conventional open plasma system using identical electrical parameters. Expanded assessment extended this comparison to Sabouraud dextrose agar and anaerobic blood agar. The tube-sealed PA-PRP showed no detectable eukaryotic microorganism or anaerobic bacterial colonies under the tested culture conditions. However, the open-activated PA-PRP produced eukaryotic microbial colonies on Sabouraud dextrose agar, although no anaerobic colonies were detected (Supplementary Fig. 2A). Furthermore, the tube-sealed PA-PRP samples were below the LAL assay detection limit, while the open-activated samples contained detectable endotoxin across replicates (Supplementary Fig. 2B). These results broadened the comparative contamination risk profile to include aerobic bacteria, eukaryotic microorganisms, anaerobic bacteria, and endotoxin. The tube-sealed design prevented direct exposure of the sample to the ambient environment during activation. For intra-articular injections, in which iatrogenic infection can result in septic arthritis and irreversible joint damage [28], this device may mitigate procedural contamination associated with open processing. These results were limited to comparative contamination risk tests under controlled experimental conditions, not pharmaceutical-grade sterility validation to support clinical product release that requires additional good manufacturing practice-level (GMP-level) testing under pharmacopeial standards. However, the results support pursuing further development of a closed-tube workflow with formal sterility assurance. A systematic characterization of the device identified parameters that provided a safe operating window: 6 W input power, 1% duty cycle, and 15 s activation. This maintained the product temperature at or below 37°C and the pH within the physiological range of 7.2–7.4. These boundaries have important biological significance. Temperatures exceeding 37°C risk protein denaturation and loss of growth factor bioactivity, while pH excursions beyond the buffering capacity of PRP indicate excessive reactive species generation with uncertain biological consequences.

An *in vitro* characterization showed increased CD63 levels and higher concentrations of TGF-β, total PDGF, and IGF-1 after plasma activation. A cytokine array also identified changes in several additional factors. Electron paramagnetic resonance (EPR) spin trapping provided qualitative evidence of singlet oxygen signals rather than hydroxyl radical signals in the activated liquid phase. In addition, a fluorescence-based analysis of PA-PRP performed 0, 1, 4, and 8 h after preparation showed a time-dependent decline in the bulk ROS signal (Supplementary Fig. 1D). This result indicated that the assay-detectable ROS signal was transient under the tested storage conditions. However, individual reactive species were not identified, and therefore, their absolute concentrations and stability were not established, and an exposure window relevant to biological activity was not defined. Plasma-activated liquids can contain short-lived ^1^O_2_, OH, and O_2_•^−^, together with longer-lived H_2_O_2_, nitrite, and nitrate, with their relative abundances dependent on the discharge and liquid conditions [1]. Thus, with the 15-s activation time and qualitative EPR signals, the species that persisted, drove platelet activation, or elicited macrophage marker changes could not be identified. The observed macrophage marker changes and growth factor release were compatible with a proposed two-component mechanism involving platelet degranulation and local immunomodulation. A related plasma-activated medium study in periodontal cells reported a shift from an inflammatory phenotype to an M2-like macrophage phenotype together with downstream regenerative effects [5]. In that model, the macrophage response was accompanied by deactivation of the protein kinase B (AKT) signaling pathway and induction of autophagy associated markers, whereas a controlled-release cold atmospheric plasma system promoted M2-associated oxidative phosphorylation and fatty acid oxidation programs along with wound repair [3]. These studies identified autophagy and metabolic reprogramming as potential downstream processes through which plasma-derived reactive species environments may couple immunomodulation to tissue repair. Although these findings arose from distinct models, they provide a rationale for testing the same effects in PA-PRP–treated joint cells. In this study, PA-PRP supplemented with 10 mM NAC attenuated the PA-PRP–associated reduction in *iNOS* expression and increase in *ARG1* expression after 24 h of macrophage culture (Supplementary Fig. 3E and F). This result identified a defined macrophage marker phenotype sensitive to ROS scavenging, thereby providing pharmacological evidence that plasma-derived reactive species contribute to the PA-PRP response. However, these findings did not identify the responsible reactive species or their persistence, the downstream signaling pathway, or macrophage-dependent effects *in vivo*. The data therefore provides a focused experimental basis for future studies combining post-activation species-resolved characterizations with analyses using selective scavengers, pathway inhibitors, macrophage depletion, and macrophage-specific perturbations. These experiments can analyze whether specific reactive species and candidate autophagy or metabolic pathways causally link PA-PRP exposure to macrophage polarization and joint outcomes.

In the DMM mouse model, PA-PRP was associated with a higher composite gait recovery index than PRP, with changes in the stride length, stride width, and paw rotation angle. The lower OARSI scores and lower MMP3, MMP9, and MMP13 levels provide a structural and molecular context for these functional observations. Human OA cartilage explants also showed higher Safranin O and Alcian blue matrix staining after PA-PRP exposure than after PRP or no treatment. This pattern suggested that the explants provided a useful preclinical model for evaluating plasma activation of PRP, complementing the use of surgical OA models and cartilage-focused phenotypes in prior PRP research [29]. These findings provide a rationale for determining whether the functional, tissue, and molecular observations can be reproduced across independent OA models and linked to defined cellular mechanisms. Gait measures are not direct measures of pain, and the gait experiments performed in this study were not designed to infer an analgesic mechanism or clinical efficacy. Our future studies will build on this phenotype through longitudinal behavioral assessments, blinded histological replication, dose and exposure optimization, and adequately powered comparisons with prespecified structural and functional endpoints.

The split-body clinical study involving three patients contributed paired procedural and short-term follow-up observations that included numerical between-knee VAS score patterns, WOMAC scores, effusion volumes, ultrasound findings, and flexion angles. Notably, no adverse events were reported at the scheduled visits. These observations inform the PA-PRP preparation and administration workflow feasibility using the device and help define outcome domains for a future study. The randomized placebo-controlled trial demonstrated the importance of an adequately powered, controlled evaluation with prespecified patient-reported and structural endpoints when assessing an intra-articular PRP product [22]. Accordingly, the week 15 VAS pattern and early changes in effusion should be interpreted as preliminary findings for guiding sample size planning and endpoint selection, rather than as evidence of a treatment effect or its mechanism. The cell viability assay and mouse organ histology results provided complementary short-term safety information. Larger randomized trials with a longer follow-up that ascertain systematic adverse events, evaluate repeated-dose, and monitor potential delayed effects of plasma-generated species can now test the clinical benefit of PA-PRP generated by our device and characterize the safety profile more fully.

Several limitations of the present study should be acknowledged. The DMM experiment used five mice per group as an exploratory study designed in accordance with the 3R principles. The selection and assessment bias was reduced by random group allocation and blinded gait and histological assessments, but they did not compensate for the limited precision associated with a small group size. A prospective power calculation was not performed, indicating that the study may be underpowered for some outcomes, particularly null findings, and that observed effect estimates may be sensitive to sampling variation. The animal results therefore provide convergent, hypothesis-generating evidence rather than definitive efficacy or mechanistic proof and should be independently replicated in larger, prospectively powered studies. The dosing schedule of weekly injections for a total of 4 weeks was selected as an exploratory regimen, and prior rodent models have differed in treatment initiation, number of injections, and treatment interval [29], [30]. Future dose-ranging studies should vary these parameters to optimize the DMM model exposure schedule, and adequately powered experiments should be performed that combine macrophage depletion and pathway inhibition designs with the direct characterization of reactive species post-activation. These experiments may clarify whether macrophages, autophagy-associated signaling, and metabolic programs causally link PA-PRP exposure to the observed gait and cartilage outcomes. The clinical study enrolled three patients in a proof-of-concept design, which does not support definitive efficacy conclusions. The weekly three-dose schedule was selected as an exploratory clinical regimen. Published PRP protocols for knee OA vary in injection number and interval [31], and the present study was not designed to identify an optimal schedule. Future randomized studies should compare prespecified dosing schedules and follow-up intervals in terms of patient-reported outcomes, structural measures, and safety. A larger sized double-blind, randomized controlled trial with extended follow-up to capture long-term structural outcomes is currently under preparation. The proof-of-concept study also did not include calcium chloride- or thrombin-activated PRP comparators. Conventional chemical activation of PRP requires additional aseptic manipulation with the introduction of an exogenous activator after the PRP is prepared, which increases the procedural complexity and contamination control, batch consistency, and product characterization requirements. In contrast, the vial wall-based DBD configuration was designed to activate PRP without adding a chemical activator to the collection tube. The efficacy advantage of this design over conventional chemical activation methods was not established as head-to-head comparisons were not performed. Future studies should directly compare the effects of plasma activation with calcium chloride- and thrombin-based activation using matched PRP products by examining growth factor release, platelet function, sterility, residual reagents, and short- and long-term intra-articular safety. Donor demographics and pre-preparation blood counts were documented to characterize the starting material; however, platelet recovery, leukocyte composition, and residual erythrocyte content were not systematically quantified in the final PRP product prior to activation. Therefore, the present study could not fully separate activation-dependent effects from variability in the PRP composition. Standardized characterizations of PRP pre- and post-activation should be incorporated in future studies. From an engineering standpoint, the current prototype with separate components has a footprint larger than ideal for point-of-care deployment. The custom prototype, comprising a programmable power supply, a high-voltage converter, a pulse controller, and a vial wall-based DBD plasma reactor, costs approximately US$4000. This prototype was designed for a greater parameter range than the 6 W, 1% duty-cycle, 15-s operating window used here. After the required operating range is confirmed, a purpose-built power supply, converter, and controller are expected to reduce the corresponding hardware cost to below US$1000. The reactor uses shaped electrodes and has a 3D-printed housing, and therefore, it has a relatively small contribution to the total cost. These estimates are based on prototype fabrication and require formal cost, manufacturability, maintenance, and quality control assessments before clinical deployment. Future iterations will integrate the power supply, high-voltage converter, pulse controller, and reactor into a compact, single-enclosure format with an expanded capacity for parallel sample processing. A potential logical next engineering step would be incorporating real-time ROS sensing into the system with closed-loop feedback control. The system could then dynamically adjust activation parameters based on real-time measurements for personalized plasma dosing titrated to individual patient characteristics, including OA severity, baseline platelet reactivity, and inflammatory status. The present results are limited to PRP activation in OA-related experimental settings. The system could potentially be extended to other musculoskeletal disorders, vaccines, cell therapies, or monoclonal antibody formulations in the future, although this was not explored or supported by the current study. Each candidate liquid therapeutic will require a dedicated evaluation of the discharge compatibility, reactive species exposure, product identity and function preservation, sterility assurance, dose control, and short-term and long-term safety before translational use can be considered.

## 4 Conclusion

In this study, we developed a vial wall-based DBD plasma activation system that activates PRP within a sealed commercial blood collection tube. Under the tested conditions, PA-PRP had increased platelet activation marker levels and growth factor concentrations relative to paired PRP, and mouse and human explant experiments provided preclinical evidence consistent with potential chondroprotective activity. The results of the microbiological assays indicated a lower contamination risk using the tube-sealed procedure than with conventional open activation systems. The cell viability, mouse observation, and three patient clinical findings provided short-term safety observations. The device therefore warrants further assessment in adequately powered trials with a standardized PRP product characterization, mechanistic validation, and longer follow-up.

## 5 Materials and methods

### 5.1 Study design

This proof-of-concept translational study included a device feasibility assessment, biological activity characterization, and exploratory preclinical and clinical studies. The five healthy PRP donors were study investigators, three men and two women, aged 36.6 ± 15.2 years. Their baseline platelet and leukocyte counts were 237.8 ± 23.5 × 10^9^/L and 4.23 ± 0.53 × 10^9^/L, respectively. The PRP used in the animal study was obtained from a 35-year-old male donor with baseline platelet and leukocyte counts of 242 × 10^9^/L and 4.13 × 10^9^/L, respectively. The three clinical participants, two men and one woman, were aged 47.7 ± 16.2 years. Two of the participants had Kellgren-Lawrence (K-L) grade 2 OA and one had K-L grade 3 OA. Their baseline platelet and leukocyte counts were 186.0 ± 18.5 × 10^9^/L and 4.53 ± 0.65 × 10^9^/L, respectively. These pre-preparation complete blood counts characterized the donor-derived starting material and did not represent the cellular composition of the final PRP product. The final PRP platelet recovery, post-preparation leukocyte composition, and residual erythrocyte content were not systematically quantified, and they are therefore not reported.

The animal experiments used female C57BL/6 mice, which were divided into four groups (n = 5 per group): sham, OA, PRP, and PA-PRP. The *ex vivo* study used cartilage explants obtained from five patients. The animal group size was selected for an exploratory feasibility study to balance the need to obtain preliminary biological data against the reduction component of the 3R principles. The refinement principle was implemented by optimizing surgical and peri-procedural care. The replacement principle was not feasible in this particular case because the gait and joint tissue outcomes required an intact OA model. Mice were randomly assigned to groups, and gait and histological outcome assessments were blinded to the group allocation. No formal prospective power calculation was performed. The small group size limited the precision of effect estimates and increased the susceptibility to sampling variation, and therefore, both positive and negative animal findings should be interpreted as hypothesis-generating rather than confirmatory. The clinical results are likewise descriptive rather than efficacy-confirming. Exact test statistics, 95% confidence intervals (CIs), degrees of freedom, and *p* values are reported when available. The clinical endpoints did not have pre-specified minimal clinically important differences.

Data collection ceased at pre-specified endpoints without interim rules. Exclusion criteria were prospectively limited to clear technical failure. No data were excluded in this study. Statistical analyses were performed using two-tailed Student's *t* tests or ANOVA with appropriate post-hoc corrections. No multiplicity correction was applied to exploratory clinical endpoints.

### 5.2 Vial wall-based DBD plasma activation device design

The complete device consists of four core components: a pulsed power supply (CTP-2000KP, Nanjing Suman Plasma Technology, China); a custom-designed DC-AC high voltage (HV) converter (1–20 kV, 1–20 kHz, integrated with voltage step-up, filtering, rectification isolation, voltage stabilization, and inversion modules and assembled by Nanjing Suman Plasma Technology, China); a custom-designed pulse controller (0.1%–100% duty cycle, assembled by Nanjing Suman Plasma Technology, China); and a self-designed vial wall-based DBD reactor, compatible with standard commercial 5–30-mL blood collection tubes. The electrical connections of the system form a complete closed-loop control circuit. The pulsed power supply outputs pulsed signals to the DC-AC HV converter, which converts and boosts the input into high-voltage pulsed output. The pulse parameter controller communicates bidirectionally with both the pulsed power supply and DC-AC HV converter, allowing the pulse frequency, duty cycle, and output voltage to be adjusted in real time. The vial wall-based DBD reactor is connected to the system ground to complete the discharge circuit.

### 5.3 Fabrication of the vial wall-based DBD reactor

The vial wall-based DBD reactor, which acts as a discharge chamber, is the core functional component of the device. Its discharge unit comprises paired stainless steel electrode structures housed in a cast cylindrical polytetrafluoroethylene protective frame, which was engineered to sustain over 1,000,000 consecutive discharge cycles without measurable corrosion or wear. The inner diameter of the discharge chamber matches the outer diameter of standard blood collection tubes. Two high-voltage electrodes are arranged coaxially along the inner wall of the chamber. After inserting the blood collection tube into the chamber, the high-voltage electrodes are pressed against the outer wall of the vial via spring-loaded supports to ensure close and uniform contact with the high dielectric vial wall. A grounded annular low-voltage electrode is installed at the mid-section of the reactor, below the high-voltage electrode pair. In this design, the wall of the blood collection tube serves as the inter-electrode dielectric barrier, which maintains the entire plasma activation reaction within the sealed blood collection tube. A threaded piston position limiter is mounted at the bottom of the reactor to adjust the insertion depth of the blood collection tube by rotating the limiter. This is used to ensure that the liquid level of PRP remains 1–5 mm below the high-voltage electrodes during activation, preventing accidental discharge into the liquid phase.

### 5.4 Platelet-rich plasma preparation

Most method optimization experiments on PRP used autologous blood from study investigators, and all clinical study samples were sourced from the Zhujiang Hospital of Southern Medical University with approval from the hospital's ethics committee and written informed consent obtained from all participating patients (Ethics No. 2026-KY-202-01). Peripheral venous whole blood was collected using commercial blood collection tubes prefilled with a separation gel and an anticoagulant (Zhuhai Longtime Biological Technology Co., Ltd.‌‌, China). Briefly, after sterile venipuncture with a sterile needle, venous blood was automatically aspirated into a blood collection tube by the tube's intrinsic negative internal pressure and then gently mixed by inverting the tube to ensure complete homogeneity between the blood and the prefilled anticoagulant. The blood collection tube was then placed in a horizontal position in a centrifuge that was symmetrically balanced, and the blood was centrifuged at 1500g for 9 min at room temperature to separate the blood components by density. After centrifugation, the red blood cells sedimented to the bottom of the blood collection tube and were separated from the upper plasma fraction by the preloaded separation gel. A concentrated platelet buffy coat formed at the interface between the separation gel and the platelet-poor plasma (PPP) supernatant. To obtain 2.5-fold concentrated PRP, a portion of the upper PPP was aspirated and discarded. The volume of the discarded PPP was adjusted to ensure that the volume of the retained plasma was approximately 40% of the initial volume of the collected whole blood to achieve a 2.5-fold increased concentration of platelets. The blood collection tube was then repeatedly inverted 180° to gently resuspend the platelet buffy coat at the gel interface in the retained plasma. The homogenized suspension was the final concentrated PRP. The PRP was retained in the original blood collection tube for subsequent activation or assays.

### 5.5 System electrical characterization and plasma diagnostics

To perform all electrical measurements, a standard commercial blood collection tube containing sterile normal saline or PRP was inserted into the reactor with the liquid level adjusted to 1 mm below the high-voltage electrodes, and the spring-loaded electrodes were confirmed to be in tight contact with the outer vial wall prior to powering on the device. The minimum sustaining power threshold of the corona inception power was determined by gradually increasing the input power from 0 W until a stable homogeneous blue discharge was visually confirmed in the gas gap above the liquid level inside the blood collection tube. The electrical characteristics of the vial wall-based DBD reactor were determined using a digital oscilloscope (TBS1102B, Tektronix, USA) with a 50-Ω sampling resistor to record the output voltage and output current waveforms in real time. To measure the output charge per discharge cycle, a 0.47-μF standard capacitor was connected in series between the low-voltage electrode and system ground, and the voltage across the capacitor was recorded to calculate the accumulated charge using the formula $P=\frac{1}{T}\int_{0}^{T}U\left(t\right)I\left(t\right)dt$ (*P*: average output power; *U(t)*: instantaneous voltage; *I(t)*: instantaneous current; *T*: integration period; *t*: time). The electrical signals were recorded after the discharge reached a steady state, and representative waveforms and Lissajous figures were collected under both the minimum sustaining power and optimized activation conditions.

### 5.6 Device compatibility testing

To evaluate the generalizability of the device across different commercially available clinical consumables, blood collection tubes from multiple mainstream brands were obtained in which the material compositions, wall thicknesses, internal pressures, and dielectric constants varied. These were tested using the characterization protocol described above. The commercially available blood collection tubes from 5 different manufacturers were anonymized to avoid potential commercial bias or conflicts of interest. All key physical parameters are provided in Supplementary Table 1 to ensure research reproducibility.

### 5.7 Optimization of activation parameters

For all parameter evaluation experiments, around 2 mL of freshly prepared PRP in standard blood collection tubes was inserted into the vial wall-based reactor, with the liquid level adjusted to 1 mm below the high-voltage electrodes as previously described. PRP samples were activated using different parameter combinations: input power of 6 and 12 W, duty cycle of 1% and 2%, and activation durations of 5, 15, 30, and 45 s. There were five independent replicates per parameter combination. Immediately after plasma activation, the temperature of the activated PRP was measured using a calibrated handheld digital thermometer (HI98501, Hanna Instruments, USA) to assess thermal accumulation, and the pH was measured by a compact micro pH electrode (InLab Ultra Micro-ISM, 30244732, Mettler Toledo, Switzerland) to evaluate acidification. The total ROS concentration in the activated PRP was quantified using a commercial ROS analysis kit (BL714A, Biosharp, China) based on the 2′,7′-dichlorofluorescin diacetate (DCFH-DA) fluorescent probe. Briefly, 100 μL of plasma-activated PRP was mixed with 100 μL of the working probe solution and incubated at 37°C for 30 min in the dark, following the manufacturer's protocol. The fluorescence intensity was measured using a microplate reader (Bio-Rad, USA) with an excitation of 480 nm and an emission of 520 nm. A standard calibration curve was constructed using gradient-diluted hydrogen peroxide solutions to convert the fluorescence intensity to an absolute ROS concentration. The optimal activation parameter window was defined as a final product temperature ≤ 37°C and a pH within the physiological range of 7.2–7.4, consistent with native PRP. All PRP samples used for parameter optimization analyses were obtained by opening the sealed vials after activation. These PRP samples were discarded as biomedical waste in accordance with institutional biosafety regulations and were not used in subsequent cell culture experiments, animal experiments, or clinical trials.

### 5.8 Device stability testing

To evaluate the long-term operational stability of the device under the optimized conditions, 50 consecutive 5-mL saline samples in identical blood collection tubes procured from a single manufacturer were activated sequentially. After each activation cycle, the total ROS concentration of the PA-PRP was measured using the analysis protocol described above. The CV of the ROS concentration of the 50 consecutive samples was calculated to quantify the device stability.

### 5.9 Contamination risk assessment of the tube-sealed activation system

After preparing the PRP, the original sealed blood collection tube containing the PRP was inserted into our vial wall-based DBD plasma activation device for tube-sealed plasma activation. The control PA-PRP was prepared in an unsealed blood collection tube, and a conventional commercial open plasma jet device was positioned 1 mm above the PRP liquid surface for open activation. Both groups were activated under identical conditions with an input power of 6 W, duty cycle of 1%, and activation durations of 5 and 15 s. Immediately after activation, 20 μL was aspirated from both the tube-sealed and open activation PA-PRP and evenly spread onto antibiotic-free LB agar plates. LB agar was prepared using LB agar powder (Thermo Fisher Scientific, 22700-025, USA) according to the manufacturer's instructions and poured into sterile Petri dishes for use. The inoculated plates were incubated at 37°C for 24 h in a constant-temperature incubator, after which the number of bacterial colonies on each plate was counted and recorded. To extend the comparative contamination risk assessment beyond aerobic bacteria, PA-PRP samples were also inoculated onto Sabouraud dextrose agar (Macklin, 771979, China) to culture eukaryotic microorganisms, including fungi and yeasts, and onto Columbia blood agar (Macklin, C999299, China) to culture anaerobic bacteria under corresponding standardized culture conditions. The endotoxin concentration of the PA-PRP was quantified using a chromogenic LAL assay.

### 5.10 Plasma-activated PRP generation

After undergoing the standard PRP preparation described previously, the original blood collection tube containing approximately 5 mL of 2.5-fold concentrated PRP (with the separation gel and a variable volume of residual red blood cells, the samples had a variable total volume of more than 5 mL) was inserted directly into the vial wall-based DBD reactor of our self-designed device. Due to the variable residual volumes of the separation gel and red blood cells, the total liquid height in the tube was not fixed. To standardize the activation condition, the liquid level was adjusted to 1 mm below the high-voltage electrode using a limit piston mounted on the bottom of the reactor. The high-voltage electrodes were positioned tightly against the outer tube wall. The PRP was activated using pre-optimized parameters (6 W input power, 1% duty cycle, and 15 s activation duration). To characterize the general types of ROS present in the activated PA-PRP liquid phase, EPR spectroscopy was used with specific spin trapping agents, 5,5-dimethyl-1-pyrroline N-oxide (DMPO, 92688, Sigma-Aldrich, USA) as a trapping agent for hydroxyl radicals, and 2,2,6,6-tetramethylpiperidine 1-oxyl (TEMPO, 214000, Sigma-Aldrich, USA) as a trapping agent for singlet oxygen.

### 5.11 Assessment of platelet activation

Platelet activation was assessed by immunofluorescence staining. PRP was divided into paired control and plasma-activated aliquots and fixed with 4% paraformaldehyde (BL539A, Biosharp, China). Samples were incubated with rabbit anti-human CD41 (1:200, ab134131, Abcam, USA) and mouse anti-human CD63 (1:200, ab271286, Abcam, USA) primary antibodies, followed by goat anti-rabbit Alexa Fluor 488 (1:500, ab150077, Abcam, USA) and goat anti-mouse Alexa Fluor 647 (1:500, ab150115, Abcam, USA) secondary antibodies. Slides were imaged on an Olympus BX53 upright fluorescence microscope (Olympus, Japan) using a 20× objective. CD63-positive platelets were expressed as a percentage of CD41-positive platelets in five randomly selected fields per sample and quantified using ImageJ version 1.52k (National Institutes of Health, USA).

### 5.12 Assessment of growth factor release

To quantify targeted growth factor levels, paired PRP and PA-PRP aliquots were analyzed using TGF-β (HM10058, Bioswamp, China), total PDGF (HM10054, Bioswamp, China), and IGF-1 (HM10399, Bioswamp, China) ELISA kits. The PDGF assay measured total PDGF, which included PDGF-AA, PDGF-AB, and PDGF-BB. Samples and standards were analyzed according to the manufacturers' instructions. The absorbance of the 3,3′,5,5′-tetramethylbenzidine color indicator was measured at 450 nm on a Bio-Rad microplate reader. Concentrations were calculated from fitted standard curves and expressed relative to the paired PRP. Global cytokine and growth factor changes were profiled using a human XL cytokine array kit (ARY026, R&D Systems, USA).

### 5.13 Macrophage and chondrocyte culture

Human chondrocytes and bone marrow-derived macrophages were provided by the Third Affiliated Hospital of Southern Medical University (Guangzhou, Guangdong, China). Both cell types were cultured in Dulbecco's modified Eagle medium (DMEM; Life Technologies, USA) supplemented with 1% penicillin-streptomycin and 10% (v/v) PA-PRP or PRP (prepared as previously described), while the control group was cultured in identical basal medium supplemented with 10% (v/v) fetal bovine serum. Macrophages were seeded at a density of 1 × 10^5^ cells per cm^2^ and chondrocytes at a density of 5 × 10^4^ cells per cm^2^. All cell cultures were maintained at 37°C in a humidified incubator with 5% CO_2_. For the ROS-quenching experiment, macrophages were cultured for 24 h with a medium supplemented with 10% (v/v) PRP, PA-PRP, or PA-PRP and 10 mM NAC (S0077, Beyotime, China).

### 5.14 Cell viability measurements

Cell viability was assessed using a 3-(4,5-dimethylthiazol-2-yl)-2,5-diphenyltetrazolium bromide assay (MTT; M5655, Sigma, USA). After 24 h of PA-PRP or PRP treatment of macrophages or 7 d of treatment of chondrocytes, MTT reagent was added to each well, followed by incubation for 4 h at 37°C. Formazan crystals were dissolved in dimethyl sulfoxide, and absorbance was measured at 490 nm using a microplate reader (Bio-Rad, USA). Cell viability was calculated relative to that of the FBS control group.

### 5.15 Gene expression analysis

For quantitative reverse-transcription PCR (RT-qPCR) analysis, total RNA was extracted from PA-PRP- or PRP-treated macrophages 24 h after treatment and from chondrocytes 7 d after treatment using TRIzol® reagent (Life Technologies, USA). RNA purity and concentration were determined using a NanoDrop One spectrophotometer (Thermo Fisher Scientific, USA). For each sample, 1 μg of total RNA was reverse-transcribed into cDNA using HiScript III All-in-one RT SuperMix (R333-01, Vazyme, China). Quantitative RT-qPCR was performed using a LightCycler 96 real-time PCR system (Roche, Switzerland) to determine the mRNA expression levels of the M1 macrophage marker *iNOS*, the M2 macrophage markers *CD206* and *ARG1*, and the chondrocyte-related genes *SRY-box transcription factor 9* (*SOX9*), *collagen type II alpha 1 chain* (*COL2A1*), and *matrix metallopeptidase 13* (*MMP13*). The forward and reverse primers used for each gene are listed in Table 5. Gene expression levels are presented as fold changes relative to the FBS control group.

**Table 5:** Primers used in gene expression analysis.

Target gene	Forward primer	Reverse primer
*iNOS*	5′-gctctacacctccaatgtgacc-3′	5′-ctgccgagatttgagcctcatg-3′
*CD206*	5′-agccaacaccagctcctcaaga-3′	5′-caaaacgctcgcgcattgtcca-3′
*ARG1*	5′-tcatctgggtggatgctcacac-3′	5′-gagaatcctggcacatcgggaa-3′
*SOX9*	5′-gaaggaccacccggattaca-3′	5′-ccttgaagatggcgttggg-3′
*COL2A1*	5′-cctggcaaagatggtgagacag-3′	5′-cctggttttccaccttcacctg-3′
*MMP13*	5′-ccttgatgccattaccagtctcc-3′	5′-aaacagctccgcatcaacctgc-3′
*GAPDH*	5′-gtctcctctgacttcaacagcg-3′	5′-accaccctgttgctgtagccaa-3′

### 5.16 Western blot

For Western blot analysis, total protein was extracted from PA-PRP- or PRP-treated macrophages after 24 h of treatment using radioimmunoprecipitation assay (RIPA) lysis buffer (R0278, Sigma, USA) supplemented with protease and phosphatase inhibitors. Proteins were denatured at 95°C for 10 min. For each sample, 20 μg of total protein was separated by sodium dodecyl sulfate-polyacrylamide gel electrophoresis and transferred to 0.22 μm Immobilon-P polyvinylidene fluoride membranes (PVDF; IPVH00010, Sigma, USA) at 4°C under a constant current of 10 mA. Membranes were incubated with primary antibodies against iNOS (M1 marker; 1:1000; A28530, ABclonal, China), arginase 1 (M2 marker; 1:1000; A25808, ABclonal, China), and α-tubulin (loading control; 1:5000; A6830, ABclonal, China), followed by a goat anti-rabbit secondary antibody (1:5000; AS014, ABclonal, China). Proteins were visualized using an Ultra High Sensitivity Enhanced Chemiluminescence Kit (ECL; HY-K1005, MedChemExpress, USA) and imaged using a 5200CE chemiluminescence imaging system (Tanon, China).

### 5.17 Alcian blue stain

For Alcian blue staining of glycosaminoglycan deposition, chondrocyte cultures were fixed with 4% paraformaldehyde (BL539A, Biosharp, China) for 15 min at room temperature 7 d after PA-PRP or PRP treatment. Fixed cells were stained with Alcian blue staining solution (TMS-010, Sigma, USA) for 15 min at room temperature in the dark, and excess stain was removed by washing with distilled water.

### 5.18 Mouse osteoarthritis model establishment

All *in vivo* experiments were approved by the Institutional Animal Care and Use Committee of the Third Affiliated Hospital of Southern Medical University (SYT2025082). Eight-week-old female C57BL/6 mice underwent destabilization of the medial meniscus (DMM) surgery or sham surgery and were then randomly allocated to the sham, OA, PRP, or PA-PRP groups (n = 5 per group). Under anesthesia, the articular capsule of the left knee was exposed. The ligament connecting the medial meniscus to the tibial plateau was transected to induce joint instability and establish the OA model. In the sham group, the joint capsule was exposed without ligament transection. Beginning 4 weeks after DMM surgery, the PRP and PA-PRP groups received 5 μL intra-articular injections once weekly for 4 consecutive weeks. This repeated weekly regimen was selected as an exploratory schedule informed by previous rodent studies of repeated weekly intra-articular PRP administration in anterior cruciate ligament transection (ACLT) rats and DMM mice [29], [30]. Mice were housed under standard specific-pathogen-free conditions, and body weight and survival were monitored throughout the experiment. Gait assessment and tissue collection were performed 2 weeks after the final injection, corresponding to week 10 after surgery.

### 5.19 Gait analysis

Gait assessment was performed at week 10 after surgery before tissue harvesting. The fore paws and hind paws were stained with blue and red ink, respectively. Mice were allowed to walk freely along a straight corridor lined with clean filter paper to generate footprints for analysis. Three valid consecutive gait cycles were selected for each mouse. Stride length, stride width, and paw rotation angle of the affected left hind limb were measured using ImageJ version 1.52k (National Institutes of Health, USA). Stride length was defined as the distance between 2 consecutive ipsilateral hind-paw footprints. The percentage change in stride length was calculated as the difference between the affected and contralateral healthy limbs divided by the stride length of the healthy limb and multiplied by 100%. Stride width was calculated as the sum of the perpendicular distances from the affected hind-paw footprint and the contralateral healthy hind-paw footprint to the body midline. The percentage change in stride width was calculated as the difference between the affected and healthy limb distances divided by the healthy limb distance and multiplied by 100%. Paw rotation angle was defined as the angle between the orientation of the hind-paw footprint and the body midline. The percentage change in paw rotation angle was calculated as the difference between the affected and contralateral healthy limb angles divided by 180° and multiplied by 100%. For each parameter, the recovery index was calculated as 100% minus the percentage change. The composite recovery score was calculated as the mean of the recovery indices for stride length, stride width, and paw rotation angle.

### 5.20 *Ex vivo* human articular cartilage explant culture

Human osteoarthritic articular-cartilage explants were prepared from discarded knee-cartilage tissue obtained from 5 patients with osteoarthritis undergoing total knee arthroplasty. All samples were obtained from the Third Affiliated Hospital of Southern Medical University with approval from the hospital ethics committee and written informed consent from all participants. Under sterile conditions, full-thickness cartilage corresponding to the residual thickness of the worn lesion was harvested from visually confirmed worn regions of the joint surface and trimmed into explants of approximately 0.3 cm^2^. Explants in the treatment groups were cultured in DMEM (Life Technologies, USA) supplemented with 1% penicillin-streptomycin and 10% (v/v) PA-PRP or PRP, whereas control explants were cultured in the same basal medium supplemented with 10% (v/v) FBS. All culture plates were maintained at 37°C in a humidified incubator with 5% CO_2_, and the medium was refreshed every 3 d throughout the culture period. After 14 d of culture, explants were harvested for histological staining and analysis.

### 5.21 Histological staining

After euthanasia, the entire left knee joint of each mouse was dissected and fixed in 4% paraformaldehyde (BL539A, Biosharp, China) overnight at 4°C. Fixed samples were decalcified in 10% ethylenediaminetetraacetic acid (EDTA; pH 7.1; 798681, Sigma, USA) for 4 weeks, dehydrated, and embedded in paraffin. Human cartilage explants were fixed in 4% paraformaldehyde (BL539A, Biosharp, China) for 24 h at room temperature, decalcified in 10% EDTA (pH 7.1; 798681, Sigma, USA) for 4 weeks, dehydrated through a graded ethanol series, and embedded in paraffin. Paraffin blocks were sectioned into 5 μm-thick slices using a microtome. Sections were deparaffinized in xylene and rehydrated through a descending ethanol series before staining.

For hematoxylin and eosin (H&E) staining, sections were stained with Mayer's hematoxylin for 3 min and eosin for 15 s using an H&E staining kit (C0105S, Beyotime, China). Stained slides were cleared with xylene and mounted for imaging.

For Safranin O-Fast Green staining, sections were stained with Weigert's iron hematoxylin, Fast Green for 5 min, and Safranin O for 5 min using a Safranin O-Fast Green kit (C0621S, Beyotime, China). Images were acquired using an Olympus BX53 microscope. For explant quantification, ImageJ version 1.52k was used to calculate the integrated red Safranin O-positive signal divided by the total stained tissue area defined by the red and green signals. This metric integrates Safranin O staining intensity with the tissue area represented in the section.

For immunohistochemical (IHC) staining, after deparaffinization, rehydration, and antigen retrieval, sections were blocked with 1% bovine serum albumin (BSA; A3608, Sigma, USA) for 1 h at room temperature. Sections were incubated overnight at 4°C with a primary anti-MMP9 antibody (1:200; Ab58803, Abcam, USA). Horseradish peroxidase-conjugated goat anti-rabbit secondary antibodies (1:500; Ab6721, Abcam, USA) were applied for 1 h at room temperature. Protein-antibody complexes were visualized with diaminobenzidine (DAB; P0203, Beyotime, China), and sections were counterstained with Mayer's hematoxylin.

For Alcian blue staining, after deparaffinization and rehydration, sections were stained with Alcian blue staining solution (TMS-010, Sigma, USA) for 20 min at room temperature in the dark and then washed with distilled water. Stained slides were cleared with xylene and mounted before imaging.

All stained slides were imaged using an Olympus BX53 bright-field microscope (Olympus, Japan).

### 5.22 Proof-of-concept split-body clinical study

This exploratory proof-of-concept clinical study was approved by the Medical Ethical Committee of Zhujiang Hospital of Southern Medical University (Ethics Approval No. 2026-KY-202-01) and registered in the Chinese Clinical Trial Registry. All participants provided consent before enrollment. Three patients with bilateral primary knee osteoarthritis of K-L grade 2–3 (mild to moderate) were enrolled in this randomized, single-blind, split-body clinical trial. Eligibility required clinical and imaging confirmation of bilateral knee OA of the same K-L grade, fulfillment of the American College of Rheumatology diagnostic criteria, and a VAS score of ≥3 despite ≥3 months of conservative treatment. For each participant, 1 knee was randomly assigned to receive an intra-articular PA-PRP injection, and the contralateral knee received a conventional PRP injection. Participants were blinded to the treatment assigned to each knee, and outcome assessors were blinded to group allocation during data collection and analysis.

Three intra-articular injections were administered once weekly. This exploratory 3-dose weekly regimen was informed by previous knee-OA studies evaluating repeated PRP injections [31]. Baseline assessment included general clinical data, VAS pain score, WOMAC score, maximum knee-flexion angle, and B-mode ultrasonography. Joint effusion was aspirated and recorded before each injection. The VAS score was recorded 7 d after the first injection, and baseline assessments were repeated at the final follow-up at week 15. Adverse events were recorded throughout the observation period.

Pain intensity was assessed using the 10-point VAS, where 0 indicates no pain and 10 indicates the worst imaginable pain. Knee function was evaluated using the total WOMAC score, which includes pain, stiffness, and physical-function domains. Maximum passive knee-flexion angle was measured by a blinded orthopedic surgeon using a standard goniometer. The volumes of joint effusion aspirated before the first and third injections were recorded to calculate the change in effusion volume after treatment. B-mode ultrasonography was performed at baseline and at the final follow-up at week 15 to qualitatively visualize changes in joint effusion. Local and systemic adverse events were recorded at each follow-up. Given the 3-participant proof-of-concept design, clinical outcomes were summarized descriptively by treatment knee and time point without inferential statistical testing.

### 5.23 Statistical analysis

Data are presented as mean ± SD unless otherwise indicated. Device-parameter data were analyzed using two-way analysis of variance (ANOVA). Paired platelet-activation and targeted growth-factor enzyme-linked immunosorbent assay measurements were analyzed using two-tailed paired *t* tests. The 3 targeted growth-factor endpoints were analyzed in parallel; the reported *p* values are unadjusted and should be interpreted accordingly. Gait outcomes were analyzed using ordinary one-way ANOVA followed by Šidák-adjusted multiple-comparisons testing within each outcome. Other multigroup animal, histological, and cell-viability comparisons were analyzed using one-way ANOVA with post hoc comparisons specified in the corresponding figure legends. Five mice were included in each animal group. The 3-participant split-body clinical component was summarized descriptively by treatment knee and time point. No inferential hypothesis testing, confidence intervals, or statistical-significance claims were used for clinical outcomes. Statistical analyses were performed using GraphPad Prism version 11.1.0 (GraphPad Software, USA). All reported nonclinical hypothesis tests were 2-sided, and the adjustment status is specified for each analysis.

## CRediT authorship contribution statement

**Shengfang Wang:** Writing – original draft, Visualization, Validation, Methodology, Investigation, Formal analysis, Conceptualization. **Zhipeng Li:** Writing – review & editing, Visualization, Validation, Investigation, Formal analysis. **Zunlei Gong:** Writing – review & editing, Visualization, Validation, Methodology, Investigation, Formal analysis. **Zengfa Deng:** Writing – review & editing, Visualization, Validation, Investigation, Formal analysis. **Zhihao Jin:** Writing – review & editing, Investigation. **Bo Pang:** Writing – review & editing, Investigation. **Jiawei Yao:** Writing – review & editing, Investigation. **Shuqin Zhang:** Writing – review & editing, Investigation. **Wenting Zhu:** Writing – review & editing, Investigation. **Jien Han:** Writing – review & editing, Investigation. **Yang Dai:** Writing – review & editing, Methodology. **Lianghao Wan:** Writing – review & editing, Methodology. **Jinglin Wan:** Writing – review & editing, Methodology. **Meijing Liu:** Writing – review & editing, Supervision, Resources, Project administration, Methodology. **Suan Tang:** Writing – review & editing, Supervision, Resources, Project administration, Methodology. **Li Jiang:** Writing – review & editing, Supervision, Resources, Project administration. **Xiaogang Wang:** Writing – review & editing, Supervision, Resources, Project administration, Funding acquisition, Conceptualization.

## Consent to participate

All participants enrolled in this study provided written informed consent for the publication of de-identified clinical data, including any clinical images presented in this manuscript, for academic publication purposes.

## Ethics approval

The proof-of-concept preliminary clinical trial and protocols used in this project were approved by Zhujiang Hospital of Southern Medical University Medical Ethical Committee (Ethics No. 2026-KY-202-01). All animal experiments in this study were reviewed and approved by the Institutional Animal Care and Use Committees (Ethical approval number SYT2025082).

## Funding information

10.13039/501100001809National Natural Science Foundation of China (No. 82350003).

## Declaration of competing interest

The vialwall-based DBD plasma reactor described in this manuscript is covered by Chinese utility model patent ZL202620123657.5. X.W., S.W., Y.D., L.W., J.W., and Z.D. are co-inventors on this patent. Y.D., L.W., and J.W. are affiliated with Nanjing Suman Plasma Technology Co., Ltd. Their contributions were limited to technical consultation, prototype design optimization, and prototype fabrication. They did not participate in clinical recruitment, clinical intervention, clinical outcome assessment, or clinical-data analysis. Commercial blood collection tubes were anonymized during compatibility testing, and the authors report no affiliation with their manufacturers.

## Data Availability

Data will be made available on request.

## Abbreviations

3R: Replacement, Reduction, and Refinement
ACLT: Anterior cruciate ligament transection
AKT: Protein kinase B
ANOVA: Analysis of variance
ARG1: Arginase 1
BSA: Bovine serum albumin
CAP: Cold atmospheric plasma
CD206: Cluster of differentiation 206
CD41: Cluster of differentiation 41
CD63: Cluster of differentiation 63
CI: Confidence interval
COL2A1: Collagen type II alpha 1 chain
CV: Coefficient of variation
DAB: Diaminobenzidine
DBD: Dielectric barrier discharge
DBP: Vitamin D-binding protein
DC-AC: Direct current-to-alternating current
DCFH-DA: 2′,7′-dichlorofluorescin diacetate
DMEM: Dulbecco's modified Eagle medium
DMM: Destabilization of the medial meniscus
DMPO: 5,5-dimethyl-1-pyrroline N-oxide
ECL: Enhanced chemiluminescence
EDTA: Ethylenediaminetetraacetic acid
ELISA: Enzyme-linked immunosorbent assay
EPR: Electron paramagnetic resonance
FBS: Fetal bovine serum
GMP: Good manufacturing practice
GND: Ground
H&E: Hematoxylin and eosin
HV: High voltage
IGF-1: Insulin-like growth factor-1
IGFBP-2: Insulin-like growth factor-binding protein-2
IGFBP-3: Insulin-like growth factor-binding protein-3
IHC: Immunohistochemical
IL-10: Interleukin 10
IL-1Ra: Interleukin 1 receptor antagonist
IL-4: Interleukin 4
iNOS: Inducible nitric oxide synthase
K-L: Kellgren-Lawrence
LAL: Limulus amebocyte lysate
LB: Luria-Bertani
MMP3: Matrix metalloproteinase 3
MMP9: Matrix metalloproteinase 9
MMP13: Matrix metalloproteinase 13
mRNA: Messenger RNA
MTT: 3-(4,5-dimethylthiazol-2-yl)-2,5-diphenyltetrazolium bromide
NAC: N-acetylcysteine
OA: Osteoarthritis
OARSI: Osteoarthritis Research Society International
PA-PRP: Plasma-activated platelet-rich plasma
PDGF: Platelet-derived growth factor
PDGF-AA: Platelet-derived growth factor-AA
PDGF-AB: Platelet-derived growth factor-AB
PDGF-BB: Platelet-derived growth factor-BB
PPP: Platelet-poor plasma
PRP: Platelet-rich plasma
PVDF: Polyvinylidene fluoride
RIPA: Radioimmunoprecipitation assay
RNS: Reactive nitrogen species
ROS: Reactive oxygen species
RT-qPCR: Reverse-transcription quantitative polymerase chain reaction
SD: Standard deviation
SOX9: SRY-box transcription factor 9
TEMPO: 2,2,6,6-tetramethylpiperidine 1-oxyl
TGF-β: Transforming growth factor-β
VAS: Visual analog scale
VEGF: Vascular endothelial growth factor
WOMAC: Western Ontario and McMaster Universities Osteoarthritis Index
