# Supplementary material for: Clinical validation of a vial wall-based dielectric barrier discharge plasma activation device for plasma-activated platelet-rich plasma preparation in osteoarthritis pain management
Source: Mater Today Bio. 2026 Oct 20;41:103766. doi: 10.1016/j.mtbio.2026.103766 (PMC13734778; doi:10.1016/j.mtbio.2026.103766)
Supplement: Supplementary file 1 [file mmc1.docx]

**Supplementary Figure 1. Detection of Reactive Species Generated by Plasma Activation**

A. Data from electron paramagnetic resonance (EPR) spectroscopy demonstrating the detection of reactive oxygen species in the plasma-activated liquid.

B. Bar graph showing the change in ROS concentration in PRP following plasma activation, as measured by a fluorescence assay kit (n = 5 independent samples; data are presented as mean ± SD; statistical analyzed by Student's *t*-test; *p* value is shown).

C. Representative data showing the Lissajous Figures during plasma activation process under activation parameters.

D. Representative data showing ROS decay in PA-PRP after preparation.

**Supplementary Figure 2. Comparative microbiological and endotoxin contamination assessment of tube-sealed and open plasma activation.**

A. Representative culture results of eukaryotic microorganisms and anaerobic bacteria comparing tube-sealed and open activation.

B. Heat map showing endotoxin concentrations in five independently prepared samples.

**Supplementary Figure 3. PA-PRP promotes macrophage polarization toward the M2 phenotype.**

A. Bar graph showing the fold change of *iNOS* (M1 macrophage marker) gene expression in macrophages cultured with FBS, PRP or PA-PRP, quantified by quantitative PCR (n = 3 independent experiments; data are presented as mean ± SD; analyzed by one-way ANOVA followed by paired Student's *t*-tests for within-group comparisons where applicable; *F* (2, 6) = 22.86, *p* = 0.0016; ** represent *p*<0.01).

B. Bar graph showing the fold change of *CD206* (M2 macrophage marker) gene expression in macrophages cultured with FBS, PRP or PA-PRP, quantified by quantitative PCR (n = 3 independent experiments; data are presented as mean ± SD; analyzed by one-way ANOVA followed by paired Student's *t*-tests for within-group comparisons where applicable; *F* (2, 6) = 197; *** represent *p*<0.001).

C. Bar graph showing the fold change of *ARG-1* (M2 macrophage marker) gene expression in macrophages cultured with FBS, PRP or PA-PRP, quantified by quantitative PCR (n = 3 independent experiments; data are presented as mean ± SD; analyzed by one-way ANOVA followed by paired Student's *t*-tests for within-group comparisons where applicable; *F* (2, 6) = 53.55; ** represent *p*<0.01).

D. Representative western blots showing protein expression of the M1 marker iNOS and M2 marker Arginase 1 in macrophages after culture with FBS, PRP or PA-PRP; α-tubulin was used as the loading control.

E. Bar graph showing the fold change of *iNOS* gene expression in macrophages cultured after ROS quenching (n = 3 independent experiments; data are presented as mean ± SD; analyzed by one-way ANOVA followed by paired Student's *t*-tests for within-group comparisons where applicable; *F* (3, 8) = 43.41; *** represent *p*<0.001).

F. Bar graph showing the fold change of *ARG-1* gene expression in macrophages cultured after ROS quenching (n = 3 independent experiments; data are presented as mean ± SD; analyzed by one-way ANOVA followed by paired Student's *t*-tests for within-group comparisons where applicable; *F* (3, 8) = 598.6; **** represent *p*<0.0001).

**Supplementary Figure 4. PA-PRP maintains chondrocyte phenotype.**

A. Bar graph showing the fold change of *SOX9* (chondrogenic marker) gene expression in chondrocytes cultured with FBS, PRP or PA-PRP, quantified by quantitative PCR (n = 3 independent experiments; data are presented as mean ± SD; analyzed by one-way ANOVA followed by paired Student's *t*-tests for within-group comparisons where applicable; *F* (2, 6) = 183.6; **** represent *p*<0.0001).

B. Bar graph showing the fold change of *COL2A1* (chondrogenic marker) gene expression in chondrocytes cultured with FBS, PRP or PA-PRP, quantified by quantitative PCR (n = 3 independent experiments; data are presented as mean ± SD; analyzed by one-way ANOVA followed by paired Student's *t*-tests for within-group comparisons where applicable; *F* (2, 6) = 25.05; ** represent *p*<0.01).

C. Bar graph showing the fold change of *MMP13* (cartilage degradation marker) gene expression in chondrocytes cultured with FBS, PRP or PA-PRP, quantified by quantitative PCR (n = 3 independent experiments; data are presented as mean ± SD; analyzed by one-way ANOVA followed by paired Student's *t*-tests for within-group comparisons where applicable; *F* (2, 6) = 56.96; *** represent *p*<0.001; **** represent *p*<0.0001).

D. Representative alcian blue staining images of chondrocytes after 7 days of culture with FBS, PRP or PA-PRP.

**Supplementary Figure 5. PA-PRP treatment alleviates articular cartilage degradation in a mouse OA model.**

A. Representative immunohistochemical staining images of MMP3 and MMP13 in knee joint cartilage from OA mice treated with PRP or PA-PRP; all images are shown at 20× magnification (scale bar = 100 μm).

B. Bar graph displays the quantitative analysis of OARSI score of knee joint from OA mice treated with PRP or PA-PRP (n=5 mice per group; data are presented as mean ± SD; statistically analyzed by one-way ANOVA, followed by paired Student's *t*-tests for within-group comparisons where applicable; *p* values are shown; *F* (3, 16) = 54.36).

**Supplementary Table 1. Compatibility with Various Blood Collection Tube Brands for Plasma Activation**

|  | Tube Wall Thickness (mm) | Intratubal Pressure (kPa) | Material | Relative Permittivity  (‌εᵣ) | Activation Parameters  (15 s) | ROS Increment (nM, n=3) |
| --- | --- | --- | --- | --- | --- | --- |
| Brand A | 1.10±0.01 | -84.91±1.65 | Glass | 4.5 | 6W, DC=1% | 99.21±5.61 |
| Brand B | 1.01±0.02 | -81.29±0.76 | Glass | 4.5 | 6W, DC=1% | 99.53±4.45 |
| Brand C | 0.98±0.02 | -84.02±1.40 | Glass | 4.5 | 6W, DC=1% | 106.02±5.39 |
| Brand D | 1.15±0.01 | -89.38±0.45 | PET | 3.8 | 6W, DC=1% | 129.26±6.19 |
| Brand E | 1.09±0.02 | -90.04±2.33 | PET | 3.8 | 6W, DC=2% | 119.32±5.78 |

Commercially available blood collection tubes from different manufacturers were tested, while anonymized to avoid potential commercial bias or conflict of interest. All key physical parameters are provided to ensure research reproducibility.
